# Reciprocal monoallelic expression of ASAR lncRNA genes controls replication timing of human chromosome 6

**DOI:** 10.1261/rna.073114.119

**Published:** 2020-06

**Authors:** Michael B. Heskett, Leslie G. Smith, Paul Spellman, Mathew J. Thayer

**Affiliations:** 1 Department of Molecular and Medical Genetics, Oregon Health & Science University, Portland, Oregon 97239, USA; 2 Department of Chemical Physiology and Biochemistry, Oregon Health & Science University, Portland, Oregon 97239, USA

**Keywords:** replication timing, *cis*-acting element, noncoding RNA

## Abstract

DNA replication occurs on mammalian chromosomes in a cell-type distinctive temporal order known as the replication timing program. We previously found that disruption of the noncanonical lncRNA genes *ASAR6* and *ASAR15* results in delayed replication timing and delayed mitotic chromosome condensation of human chromosomes 6 and 15, respectively. *ASAR6* and *ASAR15* display random monoallelic expression and display asynchronous replication between alleles that is coordinated with other random monoallelic genes on their respective chromosomes. Disruption of the expressed allele, but not the silent allele, of *ASAR6* leads to delayed replication, activation of the previously silent alleles of linked monoallelic genes, and structural instability of human chromosome 6. In this report, we describe a second lncRNA gene (*ASAR6-141*) on human chromosome 6 that when disrupted results in delayed replication timing in *cis*. *ASAR6-141* is subject to random monoallelic expression and asynchronous replication and is expressed from the opposite chromosome 6 homolog as *ASAR6*. ASAR6-141 RNA, like ASAR6 and ASAR15 RNAs, contains a high L1 content and remains associated with the chromosome territory where it is transcribed. Three classes of *cis*-acting elements control proper chromosome function in mammals: origins of replication, centromeres, and telomeres, which are responsible for replication, segregation, and stability of all chromosomes. Our work supports a fourth type of essential chromosomal element, the “Inactivation/Stability Center,” which expresses ASAR lncRNAs responsible for proper replication timing, monoallelic expression, and structural stability of each chromosome.

## INTRODUCTION

Numerous reports over the past 50+ yr have described an abnormal DNA replication phenotype affecting individual chromosomes in mitotic preparations from mammalian cells (for review, see Thayer 2012). For example, we found that certain tumor-derived chromosome translocations display a delay in replication timing (DRT) that is characterized by a >3 h delay in the initiation and completion of DNA synthesis along the entire length of individual chromosomes (Smith et al. 2001). Chromosomes with DRT also display a delay in mitotic chromosome condensation (DMC), which is characterized by an under-condensed appearance during mitosis and a concomitant delay in the mitotic phosphorylation of histone H3 (Smith et al. 2001; Chang et al. 2007). We also found that ∼5% of chromosome translocations induced by exposing human or mouse cells to ionizing radiation (IR) display DRT/DMC (Breger et al. 2004). To characterize the DRT/DMC phenotype further, we developed a Cre/loxP system that allowed us to create chromosome translocations in a precise and controllable manner (Breger et al. 2004, 2005). Using this Cre/loxP system, we carried out a screen in human cells designed to identify loxP integration sites that generate translocated chromosomes with DRT/DMC (Breger et al. 2004, 2005; Stoffregen et al. 2011; Donley et al. 2015). We found that ∼5% of Cre/loxP induced translocations display DRT/DMC (Breger et al. 2005). Therefore, ∼5% of translocations induced by two different mechanisms (IR or Cre/loxP) result in DRT/DMC.

Our Cre/loxP screen identified five cell lines that generate balanced translocations, affecting eight different autosomes, all displaying DRT/DMC (Breger et al. 2005). Characterization of two of these translocations identified discrete *cis*-acting loci that when disrupted result in DRT/DMC on human chromosomes 6 or 15 (Stoffregen et al. 2011; Donley et al. 2015). Molecular examination of the disrupted loci identified two lncRNA genes, which we named ASynchronous replication and Autosomal RNA on chromosome 6 (*ASAR6*) and on chromosome 15 (*ASAR15*) (Stoffregen et al. 2011; Donley et al. 2015). These studies defined the first *cis*-acting loci that control replication timing, monoallelic gene expression, and structural stability of individual human autosomes (Stoffregen et al. 2011; Donley et al. 2015).

The vast majority of genes on mammalian autosomes are expressed from both alleles. However, some autosomal genes are expressed preferentially from only one allele, achieving a state of “autosome pair nonequivalence” (Singh et al. 2003; Ensminger and Chess 2004). The most extreme form of differential allelic expression is often referred to as monoallelic expression, where a single allele is expressed exclusively (for review, see Gendrel et al. 2016). The differential allelic expression can arise from distinct mechanisms. For example, variation in gene expression can be heritable and has been mapped to the genomes of humans and model organisms as expression quantitative trait loci (eQTL) (Petretto et al. 2006). eQTL are genetic loci where sequence variation is associated with differential expression of one or more genes. The consequences of eQTL can be observed either in *cis* or in *trans* (Bryois et al. 2014; Signor and Nuzhdin 2018). In contrast, the differential allelic expression can also occur in the absence of DNA sequence polymorphisms and is connected to situations where there is a “programmed” requirement to regulate gene dosage or to provide exquisite specificity (for reviews, see Alexander et al. 2007; Li et al. 2012; Lin et al. 2012; Gendrel et al. 2014). One well-established form of programmed monoallelic expression occurs in a parent of origin-specific manner, and is known as genomic imprinting (for review, see Bartolomei 2009). In addition, monoallelic expression occurring in a random manner was observed from as many as 8% of autosomal genes (Gimelbrant et al. 2007; Chess 2012). One unusual characteristic of all programmed monoallelic genes is asynchronous replication between alleles (Mostoslavsky et al. 2001; Singh et al. 2003; Ensminger and Chess 2004; Schlesinger et al. 2009). This asynchronous replication is present in tissues where the genes are not transcribed, indicating that asynchrony is not dependent on transcription (Singh et al. 2003; Ensminger and Chess 2004; Donley et al. 2013, 2015). Furthermore, asynchronous replication of random monoallelic genes is coordinated with other random monoallelic genes on the same chromosome, indicating that there is a chromosome-wide system that coordinates replication asynchrony of programmed random monoallelic genes (Singh et al. 2003; Ensminger and Chess 2004; Donley et al. 2013, 2015). We use the following criteria to classify genes as being subject to programmed random monoallelic expression (PRME): (i) differential allelic expression is detected in multiple unrelated individuals, which rules out rare DNA polymorphisms in promoters or enhancers; (ii) differential expression of either allele is detected in single-cell-derived subclones from the same individual, which rules out genomic imprinting and eQTL; and (iii) asynchronous replication between alleles is present and coordinated with other random monoallelic genes on the same chromosome, indicating that the monoallelic gene is regulated by a chromosome-wide system that coordinates asynchronous replication along the chromosome pair. Using these criteria, we previously found that *ASAR6* and *ASAR15* are subject to PRME (Stoffregen et al. 2011; Donley et al. 2013, 2015).

Recent reports have described very long intergenic noncoding (vlinc) RNAs expressed in numerous human tissues (Kapranov et al. 2010; St Laurent et al. 2012, 2016). The vlincRNAs are RNA Pol II products that are nuclear, nonspliced, nonpolyadenylated transcripts of >50 Kb of contiguously expressed sequence that are not associated with protein-coding genes. The initial reports annotated 2147 human vlincRNAs from 833 samples in the FANTOM5 data set (St Laurent et al. 2013, 2016). A more recent study identified an additional 574 vlincRNAs expressed in childhood acute lymphoblastic leukemia (Caron et al. 2018). Therefore, there are currently >2700 annotated vlincRNAs that are encoded by >10% of the human genome (St Laurent et al. 2013, 2016; Caron et al. 2018). ASAR6 and ASAR15 RNAs share several characteristics with the vlincRNAs, including: RNA Pol II products, long contiguous transcripts (>50 Kb) that are nonspliced, nonpolyadenylated, and are retained in the nucleus (Stoffregen et al. 2011; Donley et al. 2013, 2015). Therefore, given these shared characteristics between ASAR6, ASAR15 and vlincRNAs, we consider the vlincRNAs as potential ASAR candidates.

In addition to monoallelic expression, ASAR6 and ASAR15 RNAs share additional characteristics, including: the RNAs are retained within the chromosome territories where they are transcribed, and they contain a high long interspersed element 1 (LINE1 or L1) content (Stoffregen et al. 2011; Donley et al. 2013, 2015). In this report, we used these “ASAR” characteristics to identify a second lncRNA gene that controls replication timing of human chromosome 6, which we designate as *ASAR6-141*. The *ASAR6-141* gene is located at ∼141 Mb of human chromosome 6, is subject to random monoallelic expression and asynchronous replication, and disruption of the expressed allele, but not the silent allele, leads to delayed replication of human chromosome 6 in *cis*. ASAR6-141 RNA, which was previously annotated as vlinc273 (St Laurent et al. 2016), is ∼185 Kb in length, contains ∼29% L1 sequences, remains associated with the chromosome 6 territory where it is transcribed, and is expressed in *trans* to the expressed allele of *ASAR6*. These observations support a model that includes reciprocal monoallelic expression of different ASAR lincRNA genes that control replication timing of homologous chromosome pairs.

## RESULTS

### Reciprocal random monoallelic expression of ASAR lncRNAs on chromosome 6

**FIGURE 1.**
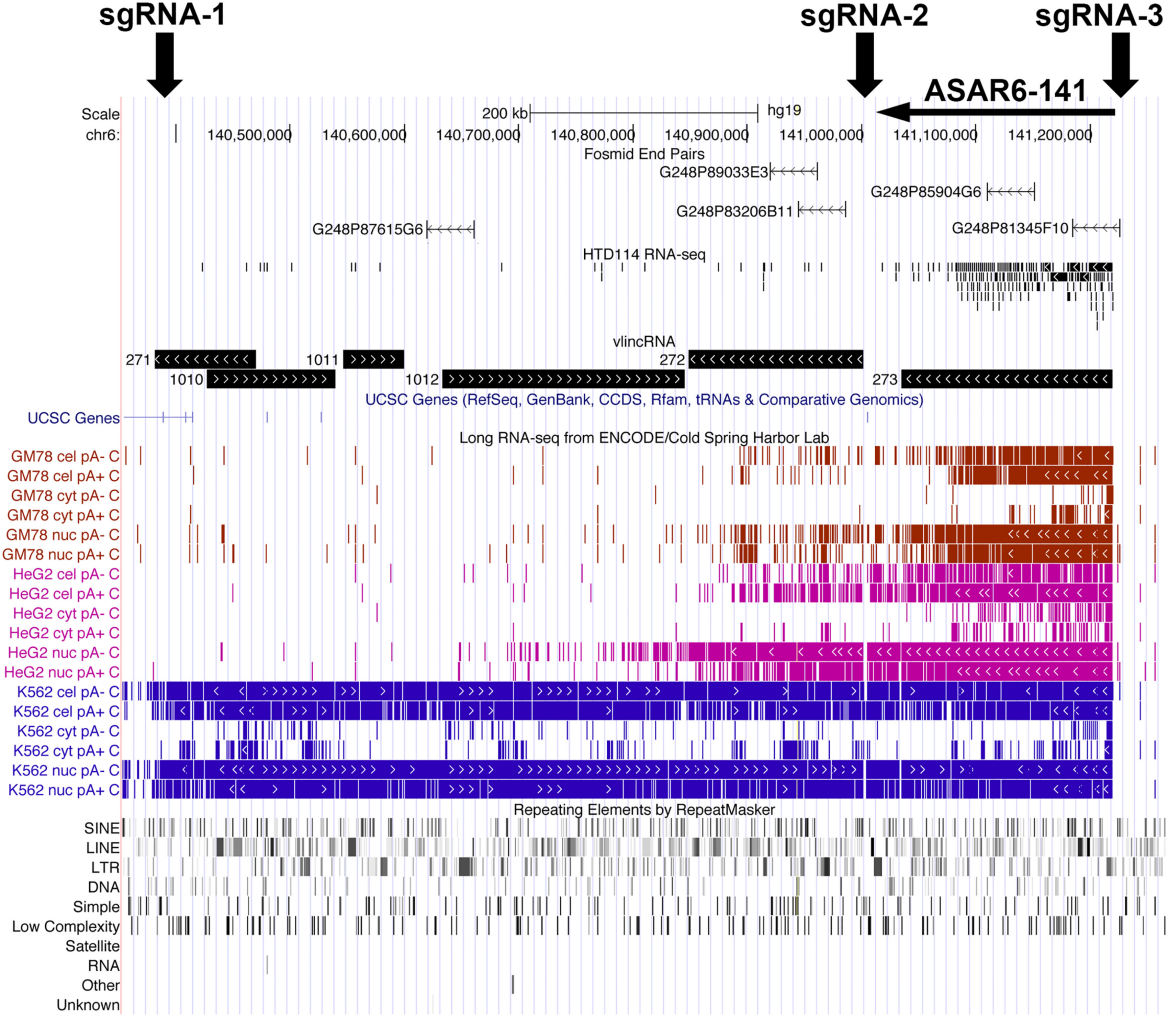
UCSC Genome Browser view of the vlinc cluster on chromosome 6 between 140.3 and 141.3 Mb. The genomic locations of *vlinc271, vlinc1010, vlinc1011, vlinc1012, vlinc272,* and *vlinc273* are illustrated using the UCSC Genome Browser (see Supplemental Table S4 for genomic locations). RNA-seq data from nuclear RNA isolated from HTD114 is shown (HTD114 RNA-seq). Long RNA-seq data from the ENCODE Project (Cold Spring Harbor Laboratory) is shown using the Contigs view. Expression from the human cell lines GM12878 (red), HepG2 (magenta), and K562 (blue) are shown. RNA from total cellular Poly A+ (cel pA+), total cellular Poly A− (cel pA−), nuclear Poly A+ (nuc pA+), nuclear Poly A− (nuc pA−), cytoplasmic Poly A+ (cyt pA+), and cytoplasmic Poly A− (cyt pA−) are shown. Also shown are the repeating elements using the RepeatMasker track. The location of five RNA FISH probes (Fosmids) that were used to detect the expression of vlinc1012 (G248P87615G6), vlinc272 (G248P89033E3 and G248P83206B11), and vlinc273 (G248P85904G6 and G248P81345F10) are shown.

With the goal of identifying nuclear RNAs with “ASAR” characteristics expressed from human chromosome 6, we carried out RNA-seq on nuclear RNA isolated from HTD114 cells. HTD114 cells are a human fibrosarcoma cell line, where we previously carried out the Cre/loxP screen that led to the identification and functional characterization of *ASAR6* and *ASAR15* (Breger et al. 2005; Stoffregen et al. 2011; Donley et al. 2015). Figure 1 shows the UCSC Genome Browser view of chromosome 6 showing the RNA-seq reads from a region, between 140.3 and 141.3 Mb, previously annotated as expressing 6 different vlincRNAs (St Laurent et al. 2016). We found that vlinc273 RNA is expressed in HTD114 cells, but vlinc271, vlinc1010, vlinc1011, vlinc1012, and vlinc272, showed little or no expression. Consistent with the characterization of vlincRNAs as being predominantly nuclear (St Laurent et al. 2016), we found that vlinc273 RNA is enriched (>100×) in the nuclear fraction in the human cell lines GM12878, HepG2, and K562 (Table 1). Figure 1 also shows the location of Fosmids used in the RNA–DNA FISH analyses (see below), the Long RNA-seq track (showing contiguous transcripts from the human cell lines, GM12878, HepG2, and K562) from ENCODE/Cold Spring Harbor, and the Repeat Masker Track showing the location of repetitive elements (see Supplemental Table S1). In addition, vlinc273 RNA was found to have significantly more L1 elements than intronic regions of similar length. For this analysis, we created a β distribution of the fraction of L1 sequences within introns by repeated random sampling of intronic regions of length equal to vlinc273 that was then used to generate an empirical *P*-value (L1 fraction within vlinc273 is 0.29, *P* = 0.013).

**TABLE 1.**
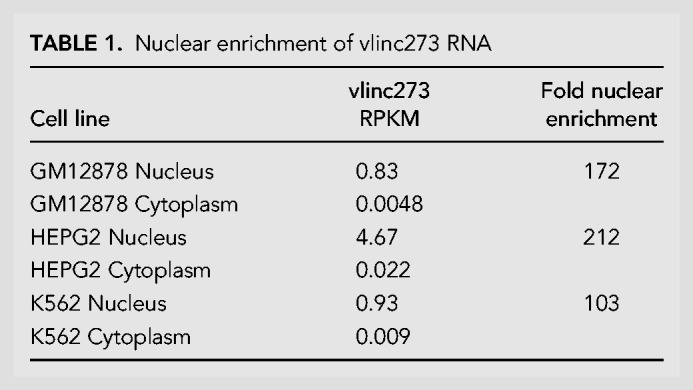
Nuclear enrichment of vlinc273 RNA

**FIGURE 2.**
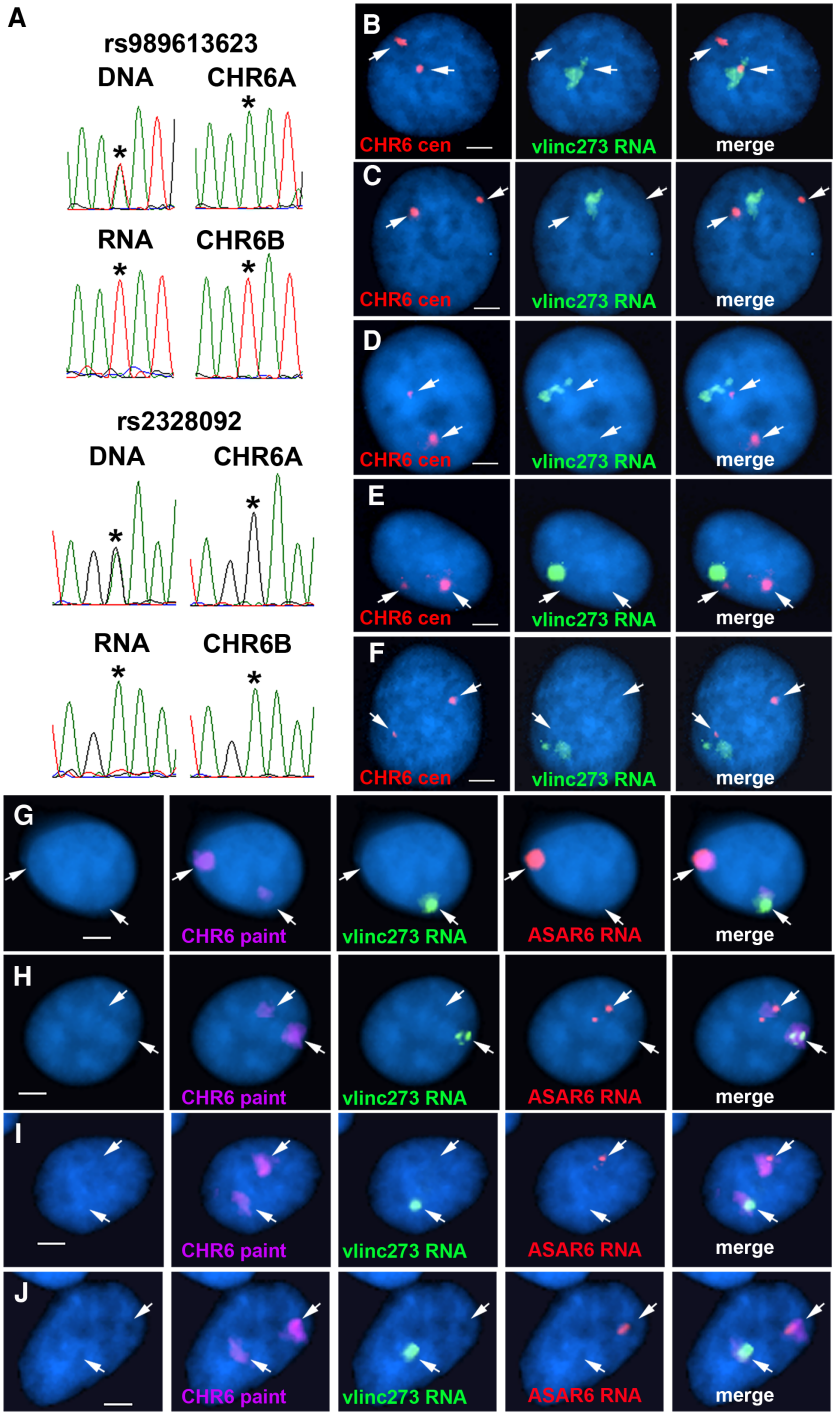
Monoallelic expression and nuclear retention of vlinc273 in HTD114 cells. (*A*) DNA sequencing traces from PCR products designed to detect SNPs rs989613623 and rs2328092 (see Supplemental Fig. S1A; Supplemental Table S2). PCRs were carried out on genomic DNAs isolated from HTD114, two monochromosomal hybrids containing the two different chromosome 6s from HTD114 [L(Hyg)-1 contains chromosome 6A (CHR6A) and expresses *ASAR6*, and L(Neo)-38 contains chromosome 6B (CHR6B) and is silent for *ASAR6* (Stoffregen et al. 2011)]. The *top* and *bottom* panels also show the sequencing traces from HTD114 cDNA (RNA). The asterisks mark the location of the heterozygous SNPs. (*B*–*F*) RNA–DNA FISH to detect vlinc273 expression in HTD114 cells. Fosmid G248P81345F10 was used as probe to detect vlinc273 RNA (green), and a chromosome 6 centromeric probe was used to detect chromosome 6 DNA (red). The nuclear DNA was stained with DAPI. Bars are 2.5 µM. (*G*–*J*) RNA–DNA FISH to detect vlinc273 and ASAR6 expression in HTD114 cells. Fosmid G248P81345F10 was used as probe to detect vlinc273 RNA (green), Fosmid G248P86031A6 was used as probe to detect ASAR6 RNA (red), and a chromosome 6 paint was used to detect chromosome 6 DNA (magenta). The nuclear DNA was stained with DAPI. Bars are 2.5 µM. Quantitation of the number of RNA FISH signals is shown in Supplemental Figure S2A and B.

One prominent characteristic of both ASAR6 and ASAR15 is monoallelic expression (Stoffregen et al. 2011; Donley et al. 2013, 2015). Therefore, to determine if the vlinc273 transcripts detected in HTD114 cells also show monoallelic expression, we used reverse-transcribed RNA as input for PCR, followed by sequencing at heterozygous SNPs. Figure 2A shows sequencing traces from two different SNPs that are heterozygous in genomic DNA, but a single allele was detected in RNA isolated from HTD114 cells, indicating that these transcripts are monoallelic (see Supplemental Fig. S1A; Supplemental Table S2). In addition, we previously generated two chromosome 6 mono-chromosomal hybrids to aid in mapping heterozygous SNPs onto the HTD114 chromosome 6 homologs (Stoffregen et al. 2011). These two hybrid cell lines are mouse L cell clones, each containing one of the two chromosome 6s from HTD114, which we arbitrarily name as CHR6A and CHR6B. Using these mono-chromosomal hybrids, we previously found that *ASAR6* is expressed from CHR6A (Stoffregen et al. 2011). Sequence traces generated from genomic DNA isolated from these two mono-chromosomal hybrids indicated that the vlinc273 transcripts are derived from CHR6B (Fig. 2A). A similar analysis of a third heterozygous SNP within vlinc273 also indicated that the RNA is monoallelic and expressed from CHR6B (Supplemental Fig. S1B; Supplemental Table S2). These results indicate that vlinc273 is expressed from the opposite chromosome 6, or in *trans*, to *ASAR6* in HTD114 cells.

Another prominent characteristic shared between ASAR6 and ASAR15 is that their RNAs remain associated with the chromosome territories where they are transcribed (Stoffregen et al. 2011; Donley et al. 2013, 2015). Therefore, we next assayed expression of vlinc273 using RNA–DNA FISH in HTD114 cells. For this analysis, we used two different Fosmid probes to detect vlinc273 RNA (see Fig. 1), plus a chromosome 6 centromeric probe to detect chromosome 6 DNA. As expected from the RNA-seq analysis, we detected the expression of vlinc273 RNA, and the RNA FISH signal remains associated with one of the chromosome 6 homologs. Figure 2B–F shows examples of this analysis and indicates that the relatively large clouds of RNA are adjacent to, or overlapping with, one of the chromosome 6 centromeric DNA signals. We detected single sites of vlinc273 RNA expression in >80% of HTD114 cells (Supplemental Fig. S2A). In addition, we used RNA–DNA FISH to detect both ASAR6 and vlinc273 RNA in combination with a chromosome 6 whole chromosome paint as probe to detect the chromosome 6 territory. Figure 2G–J shows examples of this analysis and indicates that vlinc273 and ASAR6 RNAs are detected on opposite chromosome 6 homologs. Quantitation of the RNA FISH signals indicated that >90% of the cells express vlinc273 RNA in *trans* to ASAR6 RNA (Supplemental Fig. S2B). We also note that the size of the RNA FISH signals detected by the ASAR6 and vlinc273 probes were variable, ranging from large clouds occupying the entire chromosome 6 territory, to relatively small spots of hybridization. We interpret the variability in the size of the RNA clouds to reflect the phase of the cell cycle. Thus, both ASAR6 and vlinc273 RNAs are not detectable in mitotic cells (not shown), which suggests that the RNA clouds must be reformed every cell cycle as cells exit mitosis and re-enter the G1 phase. Thus, cells with small RNA FISH signals are predicted to be in early G1, whereas cells with large RNA FISH signals are predicted to be in S or G2 phases.

### Random monoallelic expression of vlinc273

**FIGURE 3.**
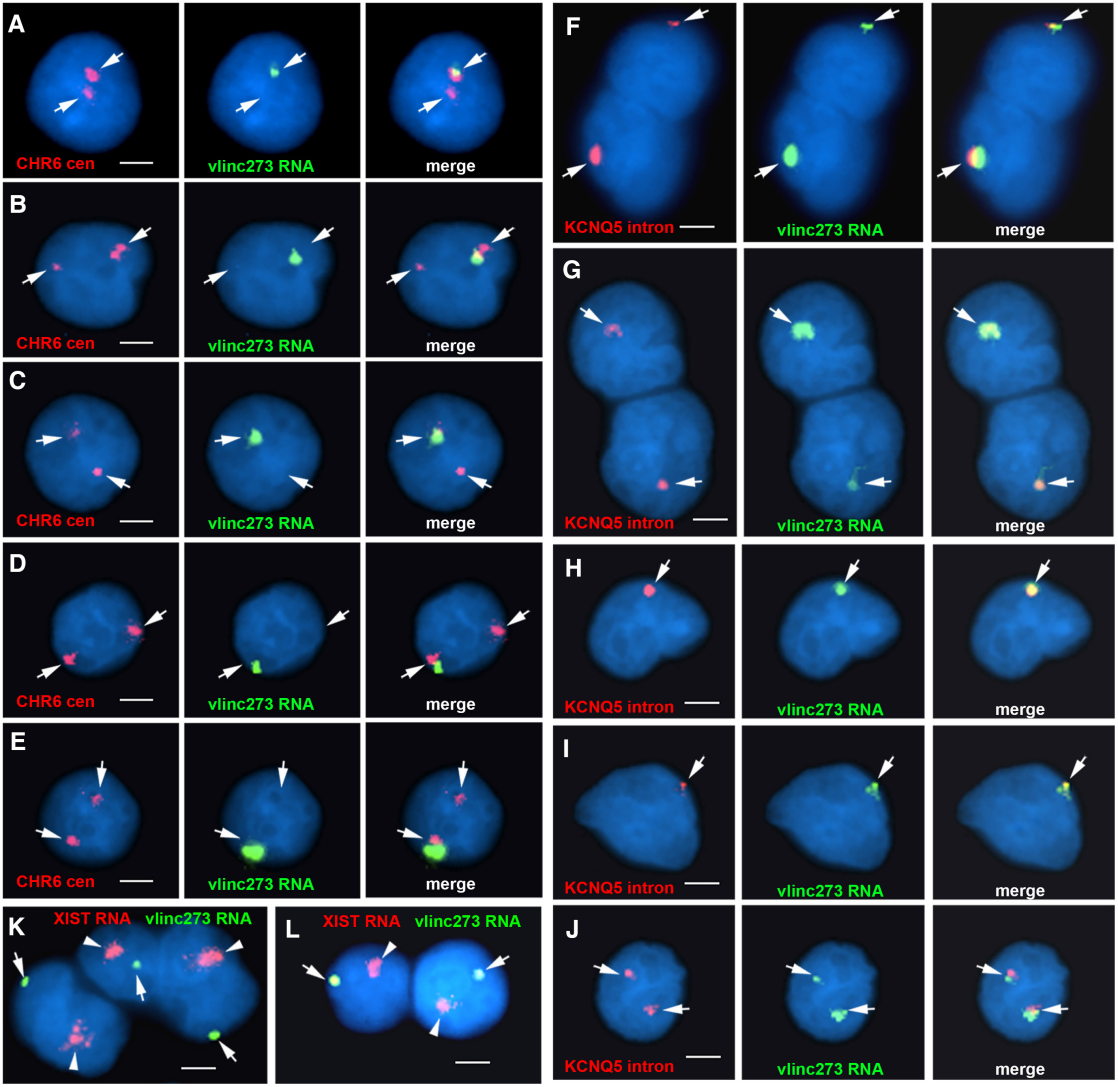
Monoallelic expression and nuclear retention of vlinc273 in EBV transformed lymphoblasts and PBLs. (*A*–*E*) RNA–DNA FISH to detect vlinc273 expression in GM12878 EBV transformed lymphocytes. Fosmid G248P81345F10 was used to detect vlinc273 RNA (green), and a chromosome 6 centromeric probe (CHR6 cen) was used to detect chromosome 6 DNA (red). (*F*–*J*) RNA FISH to detect the coordinated expression of vlinc273 and KCNQ5, a known random monoallelic gene, in PBLs. Fosmid G248P81345F10 was used to detect vlinc273 RNA (green), and Fosmid G248P80791F6 was used to detect the expression of the first intron of KCNQ5. (*K*,*L*) RNA FISH to detect the expression of vlinc273 and XIST RNAs, in female PBLs. The nuclear DNA was stained with DAPI. Bars are 2.5 µM. Quantitation of the number of RNA FISH signals per nucleus and the *cis* versus *trans* expression is shown in Supplemental Figure S2B–D.

The observations described above indicate that vlinc273 and ASAR6 RNAs are expressed from opposite chromosome 6 homologs in the clonal cell line HTD114. This monoallelic expression could be due to either DNA sequence polymorphisms within regulatory elements (i.e., eQTL), genomic imprinting, or to PRME. Therefore, to begin to distinguish between these possibilities, we determined if vlinc273 is monoallelically expressed in EBV transformed lymphoblastoid cells, which have been used extensively in the analysis of autosomal monoallelic expression in humans (Ensminger and Chess 2004; Gimelbrant et al. 2007; Stoffregen et al. 2011; Donley et al. 2013). For this analysis, we used RNA–DNA FISH to assay expression of vlinc273 in GM12878 lymphoblastoid cells. We used two different Fosmid probes to detect vlinc273 RNA, plus a chromosome 6 centromeric probe to detect DNA. For comparison, we also assayed the expression of *KCNQ5*, a gene on chromosome 6 known to be subject to PRME (Gimelbrant et al. 2007; Donley et al. 2013). Note that the RNA FISH probe for *KCNQ5* is derived from the first intron, which is ∼400 Kb in length, and this probe results in a robust RNA FISH signal. We found that vlinc273 and *KCNQ5* show similar levels of monoallelic expression with single sites of RNA hybridization in ≥84% of cells (Fig. 3A–E; Supplemental Fig. S2C).

Next, to determine if vlinc273 is monoallelic in human primary cells, we assayed the expression of vlinc273 using RNA FISH in primary blood lymphocytes (PBLs) isolated from two unrelated individuals. For comparison, we also included a probe for *KCNQ5*. We found that vlinc273 and *KCNQ5* show similar levels of monoallelic expression with single sites of RNA hybridization in ≥69% and two RNA FISH signals in ≤13% of cells (Fig. 3F–J; Supplemental Fig. S2C).

To determine if the monoallelic expression of vlinc273 is random, and not imprinted, we tested whether or not vlinc273 expression was coordinated with a gene on chromosome 6 that is known to be subject to PRME. The premise of this experiment is that PBLs are nonclonal, and individual PBL cells are anticipated to express either the paternal or the maternal allele of any gene subject to PRME at approximately equal frequencies. Thus, if vlinc273 expression is always expressed from the same or opposite chromosome 6 homolog as a known PRME gene then we can conclude that vlinc273 expression is also subject to PRME. In contrast, if vlinc273 expression is detected from the same chromosome 6 homolog as a known PRME gene in only ∼50% of cells, then we would suspect that vlinc273 expression was subject to imprinting. For this analysis, we tested if the expression of vlinc273 RNA was detected on the same or opposite chromosome, that is, either in *cis* or in *trans*, as KCNQ5 RNA, which is known to be subject to PRME (Gimelbrant et al. 2007; Stoffregen et al. 2011; Donley et al. 2013). For this analysis, we used a two-color RNA FISH assay to detect the expression of vlinc273 in combination with a probe from the first intron of *KCNQ5* on PBLs isolated from two unrelated individuals. Quantification of the number of RNA FISH signals in >100 cells indicated that vlinc273 and *KCNQ5* were expressed from the same chromosome 6 homolog in >89% of cells from both individuals (see Fig. 3F–I; Supplemental Fig. S2B). A similar assay on GM12878 lymphoblastoid cells, which are also nonclonal, indicated that vlinc273 and *KCNQ5* were expressed from the same chromosome 6 homolog in 94% of cells (Supplemental Fig. S2B). Therefore, because the PBLs and GM12878 cells are nonclonal, and *KCNQ5* expression is subject to PRME (Gimelbrant et al. 2007; Stoffregen et al. 2011; Donley et al. 2013), we conclude that the monoallelic expression of vlinc273 must also be random and therefore not imprinted. We note that we detected two sites of hybridization for both probes in ∼2% of cells (Fig. 3J). Finally, to directly compare the appearance of the RNA FISH signals detected for vlinc273 to XIST RNA expressed from the inactive X chromosome we assayed vlinc273 and XIST RNAs simultaneously in female PBLs. Figure 3K and L show the clouds of RNA detected by the vlinc273 probe in relation to the relatively larger clouds of RNA hybridization detected by the *XIST* probe.

### Asynchronous replication of *vlinc273* is coordinated on chromosome 6

**FIGURE 4.**
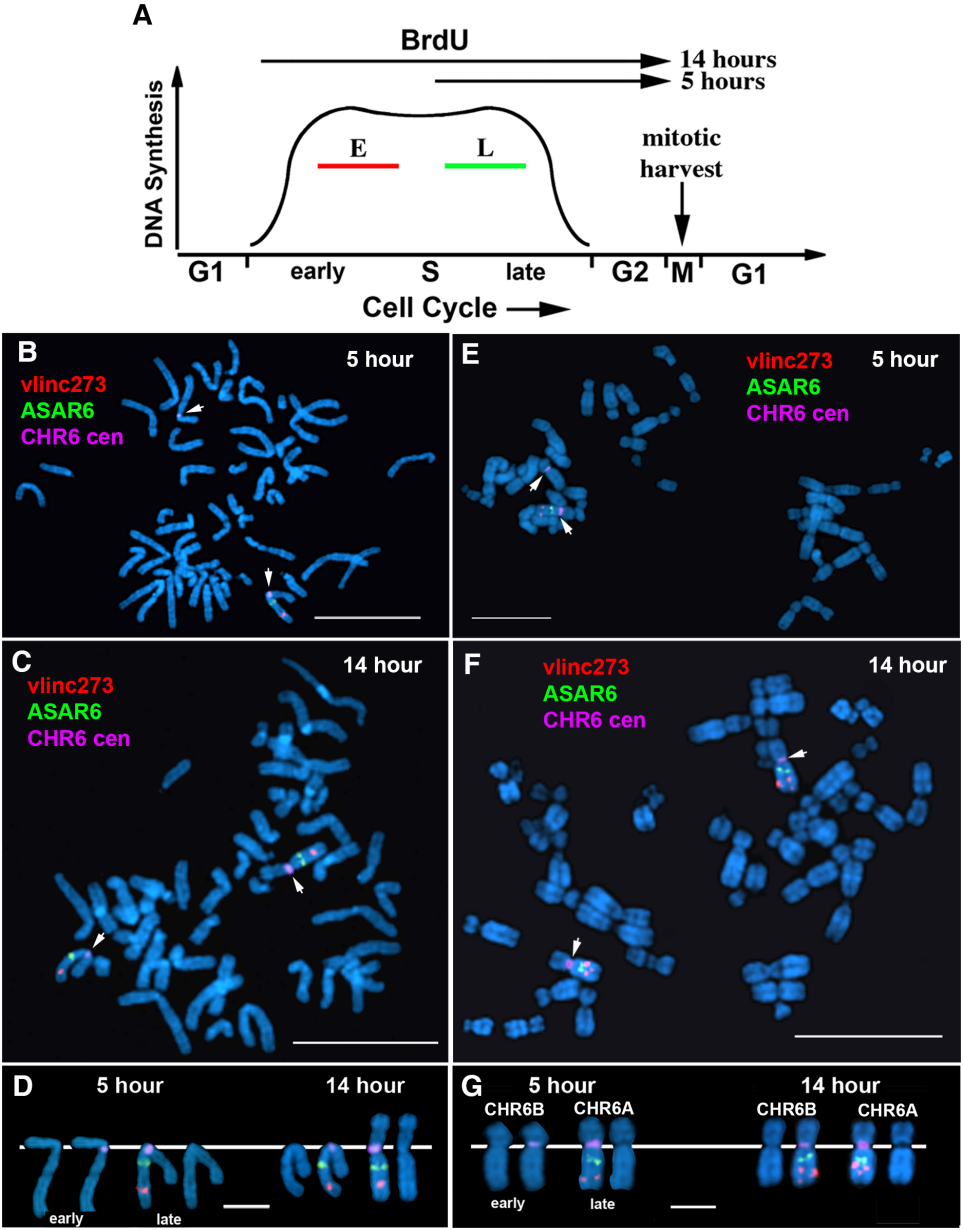
Coordinated asynchronous replication timing on chromosome 6. (*A*) Schematic representation of the ReTiSH assay. Cells were exposed to BrdU during the entire length of S phase (14 h) or only during late S phase (5 h). The ReTiSH assay can distinguish between alleles that replicate early (E) and late (L) in S phase. (*B*–*D*) Mitotic spreads from human PBLs were processed for ReTiSH and hybridized with three different FISH probes. First, each hybridization included a centromeric probe to chromosome 6 (magenta). Arrows mark the centromeric signals in panels *B* (5 h) and *C* (14 h). Each assay also included BAC probes representing *ASAR6* (RP11-374I15; green) and *vlinc273* (RP11-715D3; red). Panel *D* shows the two chromosome 6s, from both the 5 and 14 h time points, aligned at their centromeres. The *ASAR6* BAC and the *vlinc273* BAC show hybridization signals on the same chromosome 6 at the 5-h time point, and as expected hybridized to both chromosome 6s at the 14-h time point. The chromosomal DNA was stained with DAPI. (*E*–*G*) ReTiSH assay on HTD114 cells. Each ReTiSH assay included a centromeric probe to chromosome 6 (magenta). Arrows mark the centromeric signals in panels *E* (5 h) and *F* (14 h). Each assay also included BAC probes for *ASAR6* (RP11-374I15; green) and *vlinc273* (RP11-715D3; red). The chromosomal DNA was stained with DAPI. The *ASAR6* BAC and the *vlinc273* BAC show hybridization signals to one chromosome 6 homolog (CHR6A) at the 5-h time point, and as expected hybridized to both chromosome 6s at the 14-h time point.

All programmed monoallelically expressed genes share the property of asynchronous replication (Goldmit and Bergman 2004). We previously used replication timing-specific hybridization (ReTiSH) (Schlesinger et al. 2009) to assay asynchronous replication of chromosome 6 loci, including *ASAR6*. In the ReTiSH assay, cells are labeled with BrdU for different times and then harvested during mitosis (see Fig. 4A). Regions of chromosomes that incorporate BrdU are visualized by a modification of chromosome orientation-fluorescence in situ hybridization (CO-FISH), where the replicated regions (BrdU-labeled) are converted to single-stranded DNA and then hybridized directly with specific probes (Schlesinger et al. 2009). Since mitotic chromosomes are analyzed for hybridization signals located on the same chromosome in metaphase spreads, the physical distance between the loci is not a limitation of the ReTiSH assay (Schlesinger et al. 2009). We previously used this assay to show that the asynchronous replication of *ASAR6* was coordinated, either in *cis* or in *trans*, with other random monoallelic loci on human chromosome 6 (Stoffregen et al. 2011; Donley et al. 2013). For this analysis, we used PBLs and a three-color hybridization scheme to simultaneously detect the *vlinc273* locus, *ASAR6*, and the chromosome 6 centromere. The chromosome 6 centromeric probe was included to unambiguously identify both chromosome 6s. Because centromeric heterochromatin is late replicating, centromeric probes hybridize to both copies of chromosome 6 at the 14 and 5 h time points (Schlesinger et al. 2009; Donley et al. 2013). Using this assay, we found that the *vlinc273* alleles were subject to asynchronous replication that is coordinated in *cis* with *ASAR6* (Table 2; Fig. 4B–D). Therefore, because the asynchronous replication of *ASAR6* is coordinated with other random monoallelic loci on chromosome 6 (Stoffregen et al. 2011; Donley et al. 2013), we conclude that the asynchrony at the *vlinc273* locus is also part of a chromosome-wide system that coordinates the asynchronous replication of random monoallelic loci on chromosome 6.

**TABLE 2.**
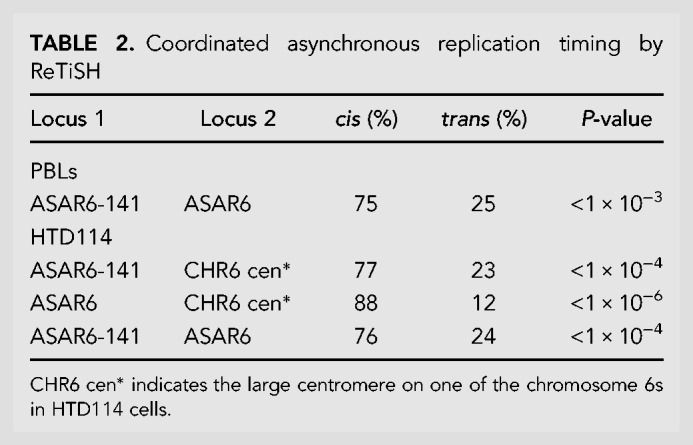
Coordinated asynchronous replication timing by ReTiSH

One shared characteristic of the *ASAR6* and *ASAR15* genes is that the silent alleles replicate before the expressed alleles on their respective chromosomes (Stoffregen et al. 2011; Donley et al. 2013, 2015). Therefore, one unanticipated result from our ReTiSH assay is that asynchronous replication of *vlinc273* and *ASAR6* is coordinated in *cis*. Thus, the earlier replicating *vlinc273* allele is on the same homolog as the earlier replicating *ASAR6* allele. Therefore, to determine if the asynchronous replication of *vlinc273* and *ASAR6* is also coordinated in *cis* in HTD114 cells, where they are expressed from opposite homologs (see Fig. 2), we analyzed the asynchronous replication of *vlinc273* and *ASAR6* using the same three-color ReTiSH assay described above (Schlesinger et al. 2009). HTD114 cells contain a centromeric polymorphism on chromosome 6, and the chromosome with the larger centromere is linked to the later replicating and expressed allele of *ASAR6* (Donley et al. 2013). We found that the asynchronous replication of *vlinc273* and *ASAR6* is coordinated in *cis* in HTD114 cells (Table 2; Fig. 4E–G). These observations are consistent with our previous finding that *ASAR6* is expressed from the later replicating allele (Donley et al. 2013; i.e., CHR6A), and indicate that *vlinc273* is expressed from the earlier replicating allele in HTD114 cells (i.e., CHR6B; see Fig. 2A). Regardless, we found that the *vlinc273* locus is subject to random monoallelic expression and asynchronous replication that is coordinated with other random monoallelic loci on chromosome 6 and therefore *vlinc273* is subject to PRME.

### Deletion of the expressed allele of *vlinc273* results in delayed replication in *cis*

To determine if the genomic region containing the vlincRNA cluster located on chromosome 6 at 140.3–141.3 Mb regulates replication timing, we used CRISPR/Cas9 to delete the entire locus in HTD114 cells (see Fig. 1; Supplemental Fig. S1A). For this analysis, we designed single-guide RNAs (sgRNAs) to unique sequences flanking the locus (see Supplemental Fig. S1A; Supplemental Table S2). We expressed sgRNA-1, sgRNA2, and sgRNA-3 in all pairwise combinations with Cas9 and screened clones for deletions using PCR primers that flank the sgRNA binding sites (see Supplemental Fig. S1A; Supplemental Table S2). Because vlinc273 expression is monoallelic in HTD114 cells, we isolated clones that had heterozygous deletions affecting either CHR6A or CHR6B. We determined which allele was deleted based on retention of the different base pairs of heterozygous SNPs located within the deleted regions (see Supplemental Figs. S1, S3, and S4; Supplemental Table S2). We also determined the deletion junctions by sequencing PCR products that span the deleted regions (see Supplemental Fig. S1A; Supplemental Table S3).

To confirm that the deletions on CHR6B removed the expressed allele of *vlinc273*, we carried out RNA–DNA FISH on cells containing deletions of either *vlinc273* alone or the entire vlincRNA cluster. Supplemental Figure S5A shows the quantitation of this analysis and indicates that vlinc273 is no longer detected in ≥86% of cells containing deletions on CHR6B. A similar analysis of cells containing deletions of ASAR6 from CHR6A indicated that ASAR6 RNA was undetectable (Supplemental Fig. S5B). In addition, to determine if deletions of the expressed allele of *vlinc273* affected the expression of *ASAR6*, and vice versa, we assayed expression of *ASAR6* in the *vlinc273* deletions on CHR6B, and expression of vlinc273 in the *ASAR6* deletions on CHR6A. Supplemental Figure S5A and B shows that vlinc273 and ASAR6 expression are not affected by deletions of the expressed alleles of their reciprocal counterparts.

**FIGURE 5.**
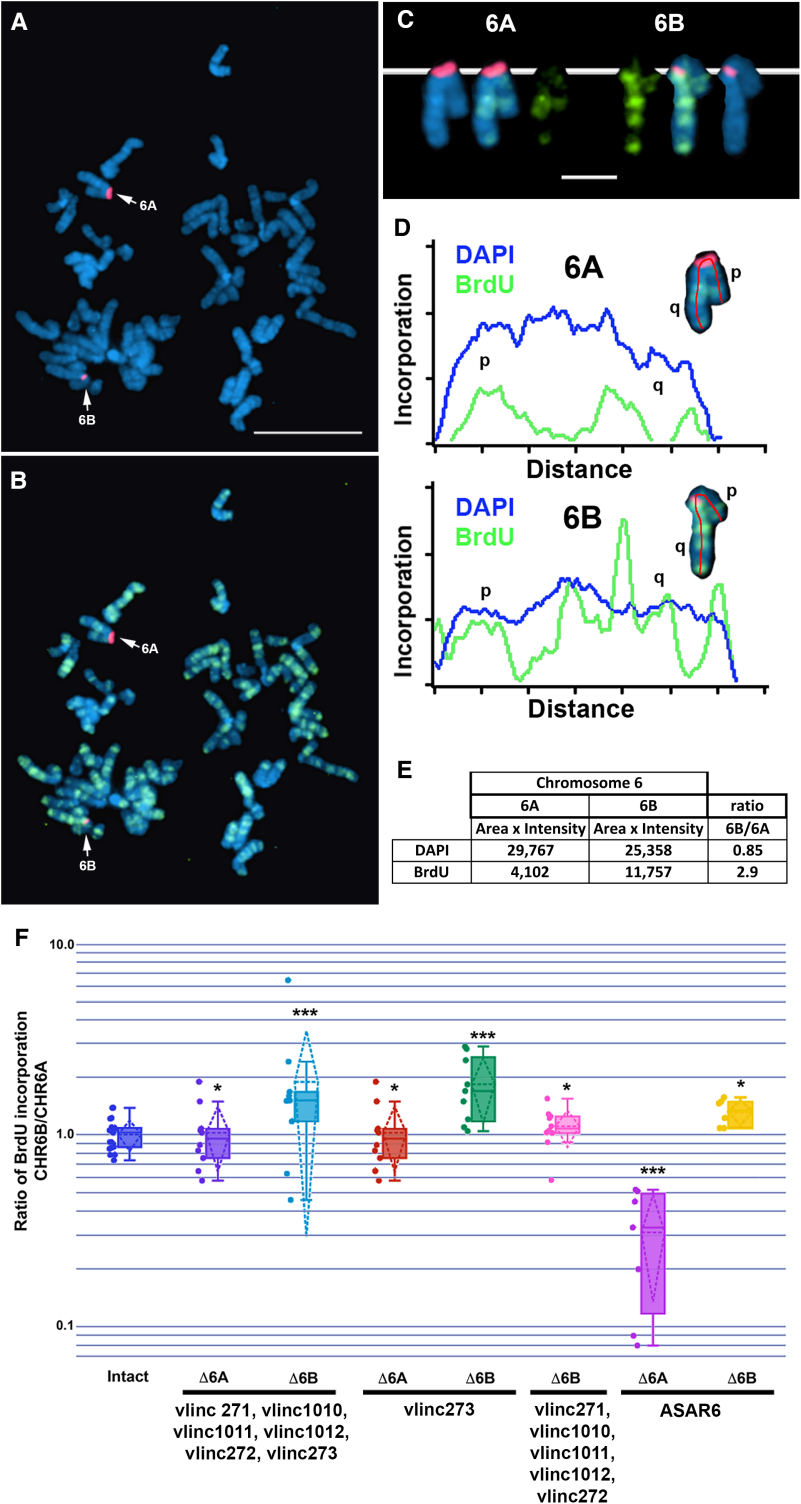
Delayed replication of chromosome 6 following disruption of *vlinc273*. (*A*,*B*) A representative mitotic spread from BrdU (green) treated HTD114 cells containing a deletion of the expressed allele of the *vlinc273* locus. Mitotic cells were subjected to DNA FISH using a chromosome 6 centromeric probe (red). The larger centromere resides on the chromosome 6 with the expressed *ASAR6* allele and the silent *vlinc273* allele (6A). (*C*) The two chromosome 6s were extracted from panels *A* and *B* and aligned to show the BrdU incorporation and centromeric signals. (*D*) Pixel intensity profiles of BrdU incorporation and DAPI staining along the (6A) and (6B) chromosomes from panel *C*. (*E*) BrdU quantification along 6A and 6B from panel *D*. (*F*) The ratio of DNA synthesis into the two chromosome 6s was calculated by dividing the BrdU incorporation in CHR6B by the incorporation in CHR6A in multiple cells. The dots show the BrdU incorporation ratios for the individual cells assayed. The box plots show the ratio of incorporation before (Intact, dark blue), and in heterozygous deletions of the entire locus (Δ6A dark purple; and Δ6B light blue), which included *vlinc271, vlinc1010, vlinc1011, vlinc1012, vlinc272,* and *vlinc273*; see maps in Figure 1 and Supplemental Figure S1A. Heterozygous deletions affecting *vlinc273* only from the silent (Δ6A orange) or expressed (Δ6B green) alleles are shown. A heterozygous deletion affecting *vlinc271, vlinc1010, vlinc1011, vlinc1012,* and *vlinc272* on CHR6B (Δ6B) is shown in pink. Also shown are heterozygous deletions affecting *ASAR6* from the expressed (Δ6A magenta) or silent (Δ6B yellow) alleles. Error bars are SD. *P*-values of <1 × 10^−4^ are indicated by (***), and *P*-values of >1 × 10^−1^ are indicated by (*), and were calculated using the Kruskal–Wallis test.

We previously found that the most sensitive assay to detect a chromosome-wide DRT involves a BrdU “terminal label” assay (Smith and Thayer 2012). This assay is distinct from the ReTiSH assay, and does not provide gene-specific information related to asynchrony at individual loci, but does provide a sensitive assay to detect chromosome-band and whole chromosome-scale changes in replication timing (Smith and Thayer 2012). Using this assay, we previously found that prior to any genetic alterations the two chromosome 6 homologs replicate synchronously in HTD114 cells (Breger et al. 2005; Stoffregen et al. 2011; Donley et al. 2013; Platt et al. 2018). In addition, we previously took advantage of a centromeric polymorphism in HTD114 cells to unambiguously distinguish between the two chromosome 6 homologs (see Fig. 4E–G; Donley et al. 2013; Platt et al. 2018). The chromosome 6 with the larger centromere is linked to the expressed allele of *ASAR6* (Donley et al. 2013; CHR6A), and therefore the expressed allele of *vlinc273* is linked to the chromosome 6 with the smaller centromere (CHR6B; see Fig. 5; Supplemental Fig. S6). For this replication timing assay, cultures were incubated with BrdU for 5.5 h and mitotic cells harvested, processed for BrdU incorporation and subjected to FISH using a chromosome 6 centromeric probe. As expected, prior to disruption of the vlinc cluster, CHR6A and CHR6B incorporate comparable levels of BrdU (Fig. 5F). In contrast, cells containing a deletion of the entire vlincRNA cluster on the CHR6B allele contain significantly more BrdU incorporation into CHR6B than in CHR6A (Fig. 5A–E). Quantification of the BrdU incorporation in multiple cells indicated that deletion of the CHR6B allele, which contains the expressed allele of *vlinc273*, results in significantly more BrdU incorporation into CHR6B (Fig. 5F). This is in contrast to cells containing a deletion of the vlincRNA cluster from the CHR6A, which is silent for all 6 vlincRNAs, where the BrdU incorporation is comparable between CHR6A and CHR6B (Fig. 5F). These results indicate that deletion of the vlincRNA cluster on CHR6B results in delayed replication of the entire chromosome in *cis*. In addition, replication timing analysis of heterozygous deletions encompassing only the *vlinc273* locus (using sgRNA-2 and sgRNA-3) indicated that deletion of the expressed allele (CHR6B), but not the silent allele (CHR6A), resulted in delayed replication of chromosome 6 (Fig. 5F). Finally, deletion of the *vlinc271, vlinc1010, vlinc1011, vlinc1012,* and *vlinc272* loci (using sgRNA-1 and sgRNA-2) on CHR6B, did not result in delayed replication of chromosome 6 (Fig. 5F). For an additional comparison, we included the chromosome 6 replication timing data from HTD114 cells containing heterozygous deletions of *ASAR6* on the expressed allele (CHR6A) and on the silent allele (CHR6B; Fig. 5F; also see Supplemental Fig. S6). Taken together these results indicate that deletion of the expressed allele of *vlinc273* results in delayed replication of chromosome 6 in *cis*, and because *vlinc273* also displays PRME, the *vlinc273* locus is an ASAR. Because *vlinc273* is the second ASAR identified on human chromosome 6, and is located at ∼141 Mb, we designate this gene as *ASAR6-141*.

## DISCUSSION

**FIGURE 6.**
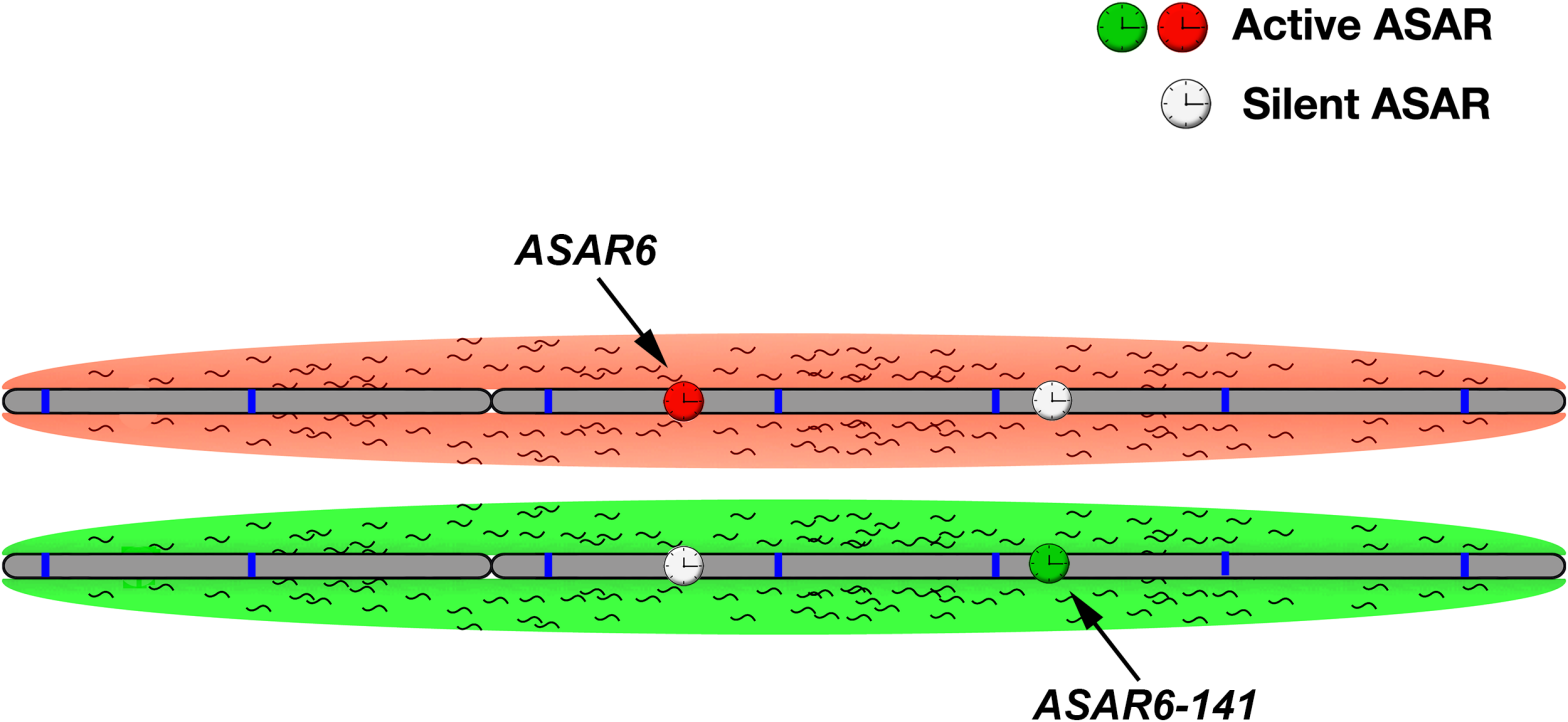
“ASAR” model of replication timing on chromosome 6. The two homologs of human chromosome 6 are shown (gray) with hypothetical origins of replication depicted as blue bars. Expression of *ASAR6* and *ASAR6-141* genes is monoallelic, resulting in a reciprocal expression pattern with an expressed or active ASAR (green or red clock) and a silent or inactive ASAR (white clock) on each homolog. The red and green clouds surrounding the chromosomes represent “ASAR” RNA expressed from the different active “ASARs” on each homolog.

Chromosome associated lncRNAs have become well established as regulators of chromosome-scale replication timing, gene expression and structural stability (for reviews, see Thayer 2012; Galupa and Heard 2018). In this report, we identified a second chromosome 6 lncRNA gene, *ASAR6-141*, that when disrupted results in delayed replication timing of the entire chromosome in *cis*. *ASAR6* and *ASAR6-141* are subject to PRME, are expressed from opposite chromosome 6 homologs, and disruption of the expressed alleles, but not the silent alleles, leads to delayed replication timing of human chromosome 6 in *cis*. ASAR6 and ASAR6-141 RNAs share certain characteristics, including RNA Pol II products that are nonspliced, nonpolyadenylated, contain a high L1 content and remain associated with the chromosome territories where they are transcribed. Taken together our results indicate that the replication timing of human chromosome 6 is regulated by the reciprocal monoallelic expression of two different ASAR lncRNA genes (Fig. 6).

We previously found that *ASAR6* controls the transcriptional activity of nearby monoallelic genes in *cis* (Stoffregen et al. 2011). Thus, we found that genetic disruption of the expressed allele of *ASAR6* leads to the transcriptional activation of the previously silent allele of the protein-coding gene *FUT9* (Stoffregen et al. 2011). Our work supports a model where the reciprocal monoallelic expression of ASAR genes controls the transcriptional activity of linked protein-coding genes in *cis*. Human chromosome 6 contains numerous random monoallelic genes, including *KCNQ5*, an olfactory receptor cluster at 6p22.1, and the HLA locus (Gimelbrant et al. 2007). Interestingly, loss of function mutations in *KCNQ5* have been shown to cause autosomal dominant mental retardation-46 (MRD46) (Lehman et al. 2017). Taken together, these observations raise the possibility that PRME of protein-coding genes can cause an autosomal dominant phenotype in individuals with heterozygous loss of function mutations due to the exclusive expression of the loss of function allele, creating the null phenotype, in 50% of cells. However, whether or not PRME contributes to autosomal dominant phenotypes in humans remains to be established. In addition, chromosome 6 also contains numerous imprinted genes, for example, *IGF2R*, *PLAGL1*, and *HYMAI* (Luedi et al. 2007). Whether or not ASAR genes interact with imprinted loci is currently not known.

One hallmark of genes that are subject to PRME is coordination in the asynchronous replication between alleles (Singh et al. 2003; Ensminger and Chess 2004; Donley et al. 2013, 2015). This coordination can be either in *cis*, that is, the early replicating alleles of two genes are always on the same homolog; or in *trans*, that is, the early replicating alleles are always on opposite homologs (Donley et al. 2013). In this report, we found that the asynchronous replication of *ASAR6-141* is coordinated in *cis* with *ASAR6*. This observation is consistent with our previous findings that human chromosome 6 contains loci that display random asynchronous replication that is coordinated both in *cis* and in *trans*, that some of these asynchronous loci are separated by >100 Mb of genomic DNA, and that the coordinated loci are on either side of the centromere (Stoffregen et al. 2011; Donley et al. 2013). One important unanswered question about the asynchronous replication that occurs at monoallelic genes is whether or not there is a difference in origin usage on the two alleles. Detecting replication initiation events is arguably the biggest obstacle in the human replication field. The reason why we understand so little about human DNA replication is because of the large number of sites that can serve as replication origins and their stochastic usage (Fragkos et al. 2015; Dileep and Gilbert 2018). The existing ensemble methods used to identify origins suffer from a low signal-to-noise ratio, due to the stochastic selection of origins. Single-molecule methods are needed to understand such a stochastic process, and these methods are currently too low throughput to be effective. Regardless, it will be interesting to determine if the two alleles of monoallelic loci use the same or different origins, and whether or not chromosomes with delayed replication timing also have altered origin usage.

Asynchronous replication of random monoallelic genes is an epigenetic mark that appears before transcription and is thought to underlie the differential expression of the two alleles of identical sequence (Mostoslavsky et al. 2001). Therefore, because the asynchronous replication at PRME genes is coordinated along each chromosome, the expression pattern of PRME genes is also anticipated to be coordinated, that is, in *cis*- always expressed from the same homolog; or in *trans*- always expressed from opposite homologs. We previously found that *ASAR6* and *ASAR15* are expressed from the later replicating alleles (Donley et al. 2013, 2015). In contrast, the *FUT9* protein-coding gene, which is closely linked to *ASAR6* (see Supplemental Fig. S7), is expressed from the early replicating allele (Donley et al. 2013, 2015). Therefore, PRME genes can be expressed from either the early or the late replicating alleles. One unanticipated result from our allelic expression and asynchronous replication assays described here is that *ASAR6-141* is expressed from the early replicating allele, which is the first example of an ASAR that is expressed from the early allele. Nevertheless, we found that disruption of the expressed allele, but not the silent allele of *ASAR6-141* results in delayed replication of chromosome 6, indicating that expression and not asynchronous replication is a critical component of ASAR function. This conclusion is consistent with our previous observation that ASAR6 RNA mediates the chromosome-wide effects of *ASAR6* forced expression (Platt et al. 2018). Therefore, the role of asynchronous replication at ASAR loci may serve as a mechanism to help establish which allele will be transcribed. Thus, the epigenetic mark that establishes early and late replication between the two alleles of PRME genes may function to establish asymmetry between alleles, and then depending on the regulatory promoter/enhancer elements at different PRME genes either the early or late replicating allele will be transcribed.

One striking feature of both *ASAR6* and *ASAR15* is that they contain a high density of L1 retrotransposons, constituting ∼40% and ∼55% of the expressed sequence, respectively (Stoffregen et al. 2011; Donley et al. 2015). L1s were first implicated in monoallelic expression when Dr. Mary Lyon proposed that L1s represent “booster elements” that function during the spreading of X chromosome inactivation (Lyon 1998, 2003). In humans, the X chromosome contains ∼27% L1 derived sequence while autosomes contain ∼13% (Bailey et al. 2000). In addition, L1s are present at a lower concentration in regions of the X chromosome that escape inactivation, supporting the hypothesis that L1s serve as signals to propagate inactivation along the X chromosome (Bailey et al. 2000). Further support for a role of L1s in monoallelic expression came from the observation that L1s are present at a relatively high local concentration near both imprinted and random monoallelic genes located on autosomes (Allen et al. 2003). L1s have also been linked to DNA replication timing from the observation that differentiation-induced replication timing changes are restricted to AT rich isochores containing high L1 density (Hiratani et al. 2004). Another potential link between L1s and DNA replication is the observation that ∼25% of origins in the human genome were mapped to L1 sequences (Bartholdy et al. 2015). While this observation is suggestive of a relationship between origins and L1s, it is not clear what distinguishes L1s with origin activity from L1s without (Bartholdy et al. 2015).

We previously proposed a model in which the antisense L1 sequences function to suppress splicing, and to promote the stable association of the RNA with the chromosome territories where they are transcribed (Platt et al. 2018). Consistent with this interpretation is the finding that a de novo L1 insertion, in the antisense orientation, into an exon of the mouse *Nr2e3* gene results in inefficient splicing, accumulation of the transcript to high levels, and retention of the transcript at the mutant *Nr2e3* locus (Chen et al. 2006). In addition, a more recent study found that the antisense strand of L1 RNA functions as a multivalent “hub” for binding to numerous nuclear matrix and RNA processing proteins, and that the L1 antisense RNA binding proteins repress splicing and 3′ end processing within and around the L1 sequences (Attig et al. 2018). We recently used ectopic integration of transgenes and CRISPR/Cas9-mediated chromosome engineering and found that L1 sequences, oriented in the antisense direction, mediate the chromosome-wide effects of *ASAR6* and *ASAR15* (Platt et al. 2018). In addition, we found that oligonucleotides targeting the antisense strand of the one full-length L1 within ASAR6 RNA restored normal replication timing to mouse chromosomes expressing an *ASAR6* transgene. These results provided the first direct evidence that L1 antisense RNA plays a functional role in replication timing of mammalian chromosomes (Platt et al. 2018). Because ASAR6-141 RNA also contains numerous L1 sequences oriented in the antisense direction (Supplemental Table S1), we anticipate that the antisense L1 sequences within ASAR6-141 RNA will likely represent the functional elements.

The vlincRNAs were identified as RNA transcripts of >50 Kb of contiguous RNA-seq reads that have no overlap with annotated protein-coding genes (St Laurent et al. 2016). The vlincRNAs were identified from the FANTOM5 cap analysis of gene expression (CAGE) data set, indicating that the vlincRNAs contain 5′ caps and consequently represent RNA Pol II transcripts (St Laurent et al. 2016). We previously found that *ASAR6* and *ASAR15* are also transcribed by RNA Pol II (Stoffregen et al. 2011; Donley et al. 2015). ASAR6-141 RNA shares certain characteristics with ASAR6 and ASAR15 RNAs that distinguish them from other canonical RNA Pol II lncRNAs. Thus, even though ASAR6-141, ASAR6, and ASAR15 RNAs are RNA Pol II products they show little or no evidence of splicing or polyadenylation and remain associated with the chromosome territories where they were transcribed (see Figs. 1–3; Stoffregen et al. 2011; Donley et al. 2015; St Laurent et al. 2016). Our work supports a model where all mammalian chromosomes express “ASAR” genes that encode chromosome associated lncRNAs that control the replication timing program in *cis*. In this model, the ASAR lncRNAs function to promote proper chromosome replication timing by controlling the timing of origin firing. In addition, because both *ASAR6*, and *ASAR6-141* are monoallelically expressed, our model includes expression of different ASAR genes from opposite homologs (see Fig. 6; Platt et al. 2018).

We previously found that ∼5% of chromosome translocations, induced by two different mechanisms (IR and Cre/loxP) display DRT/DMC (Breger et al. 2004, 2005). Because ∼5% of translocations display DRT/DMC and only one of the two translocation products has DRT/DMC (Breger et al. 2005), indicates that ∼2.5% of translocation products display DRT/DMC (Breger et al. 2004, 2005; Stoffregen et al. 2011; Donley et al. 2015). Taken with the observation that the translocations that display DRT/DMC have disrupted ASAR genes (Stoffregen et al. 2011; Donley et al. 2015), these observations suggest that ∼2.5% of the genome is occupied by ASARs. There are currently >2700 annotated human vlincRNAs, and they are expressed in a highly cell-type-specific manner (St Laurent et al. 2013, 2016; Caron et al. 2018). Because many of the vlincRNAs are encoded by regions of the genome that do not overlap with protein-coding genes, many of the vlincRNAs contain a high density of repetitive elements, including L1s (see Fig. 1; Supplemental Table S1 for examples). In this report, we found that the genomic region annotated as *vlinc273* has all of the physical and functional characteristics that are shared between *ASAR6* and *ASAR15*, and therefore *vlinc273* is an ASAR (designated here as *ASAR6-141*). In addition, while ASAR6 RNA was not annotated as a vlincRNA in any previous publication, our RNA-seq data from HTD114 cells indicates that ASAR6 RNA has all of the characteristics of a vlincRNA (see Supplemental Fig. S7). Furthermore, we note that there are two annotated vlincRNAs (vlinc253 and vlinc254) that map within the ∼1.2 Mb domain of asynchronous replication that we previously associated with the *ASAR6* locus (Supplemental Fig. S7; Donley et al. 2013). Therefore, *vlinc253* and *vlinc254* display asynchronous replication that is coordinated with *ASAR6*, *ASAR6-141*, and all other PRME genes on human chromosome 6 (see Donley et al. 2013). Taken together, these observations raise the intriguing possibility that these other vlincRNAs are also ASARs. Finally, the clustering of vlincRNA genes with ASAR characteristics, and their apparent tissue-restricted expression patterns (see Fig. 1; Supplemental Fig. S7), supports a model in which each autosome contains clustered ASAR genes, and that these ASAR clusters, expressing different ASAR transcripts in different tissues, function as “Inactivation/Stability Centers” that control replication timing, monoallelic gene expression, and structural stability of each chromosome.

## MATERIALS AND METHODS

### Cell culture

HTD114 cells are a human *APRT* deficient cell line derived from HT1080 cells (Zhu et al. 1994), and were grown in DMEM (Gibco) supplemented with 10% fetal bovine serum (Hyclone). GM12878 cells were obtained from ATCC and were grown in RPMI 1640 (Life Technologies) supplemented with 15% fetal bovine serum (Hyclone). PBLs were isolated after venipuncture into a Vacutainer CPT (Becton Dickinson) per the manufacturer's recommendations and grown in 5 mL RPMI 1640 (Life Technologies) supplemented with 10% fetal bovine serum (Hyclone) and 1% phytohemagglutinin (Life Technologies). All cells were grown in a humidified incubator at 37°C in a 5% carbon dioxide atmosphere.

### RNA-seq

Nuclei were isolated from HTD114 cells following lysis in 0.5% NP40, 140 mM NaCl, 10 mM Tris–HCl (pH 7.4), and 1.5 mM MgCl_2_. Nuclear RNA was isolated using TRIzol reagent using the manufacturer's instructions, followed by DNase treatment to remove possible genomic DNA contamination. RNA-seq was carried out at Novogene. Briefly, ribosomal RNAs were removed using the Ribo-Zero kit (Illumina), RNA was fragmented into 250–300 bp fragments, and cDNA libraries were prepared using the Directional RNA Library Prep Kit (NEB). Paired-end sequencing was done on a NovaSeq 6000. Triplicate samples were merged and aligned to the human genome (hg19) using the STAR aligner (Dobin et al. 2013) with default settings. Duplicate reads and reads with map quality below 30 were removed with SAMtools (Li et al. 2009).

### DNA FISH

Mitotic chromosome spreads were prepared as described previously (Smith et al. 2001). After RNase (100 µg/mL) treatment for 1 h at 37°C, slides were washed in 2× SSC and dehydrated in an ethanol series and allowed to air dry. Chromosomal DNA on the slides was denatured at 75°C for 3 min in 70% formamide/2× SSC, followed by dehydration in an ice-cold ethanol series and allowed to air dry. BAC and Fosmid DNAs were labeled using nick translation (Vysis, Abbott Laboratories) with Spectrum Orange-dUTP, Spectrum Aqua-dUTP or Spectrum Green-dUTP (Vysis). Final probe concentrations varied from 40 to 60 ng/µL. Centromeric probe cocktails (Vysis) and/or whole chromosome paint probes (Metasystems) plus BAC or Fosmid DNAs were denatured at 75°C for 10 min and prehybridized at 37°C for 10 min. Probes were applied to denatured slides and incubated overnight at 37°C. Post-hybridization washes consisted of one 3-min wash in 50% formamide/2× SSC at 40°C followed by one 2-min rinse in PN (0.1 M Na_2_HPO_4_, pH 8.0/2.5% Nonidet NP-40) buffer at RT. Coverslips were mounted with Prolong Gold antifade plus DAPI (Invitrogen) and viewed under UV fluorescence (Olympus).

### ReTiSH

We used the ReTiSH assay essentially as described (Schlesinger et al. 2009). Briefly, unsynchronized, exponentially growing cells were treated with 30 μM BrdU (Sigma) for 6 or 5 and 14 h. Colcemid (Sigma) was added to a final concentration of 0.1 μg/mL for 1 h at 37°C. Cells were trypsinized, pelleted by centrifugation at 1000 rpm, and resuspended in prewarmed hypotonic KCl solution (0.075 M) for 40 min at 37°C. Cells were pelleted by centrifugation and fixed with methanol–glacial acetic acid (3:1). Fixed cells were dropped gently onto wet, cold slides and allowed to air dry. Slides were treated with 100 μg/mL RNase A at 37°C for 10 min. Slides were rinsed briefly in H_2_O followed by fixation in 4% formaldehyde at room temperature for 10 min. Slides were incubated with pepsin (1 mg/mL in 2N HCl) for 10 min at 37°C, and then rinsed again with H_2_O and stained with 0.5 μg/μL Hoechst 33258 (Sigma) for 15 min. Slides were flooded with 200 μL 2× SSC, coverslipped and exposed to 365 nm UV light for 30 min using a UV Stratalinker 2400 transilluminator (Stratagene). Slides were rinsed with H_2_O and drained. Slides were incubated with 100 μL of 3 U/μL of ExoIII (Fermentas) in ExoIII buffer for 15 min at 37°C. The slides were then processed directly for DNA FISH as described above, except with the absence of a denaturation step. *ASAR6* DNA was detected with BAC RP11-767E7, and *ASAR6-141* DNA was detected with BAC RP11-715D3.

### RNA–DNA FISH

Cells were plated on glass microscope slides at ∼50% confluence and incubated for 4 h in complete media in a 37°C humidified CO_2_ incubator. Slides were rinsed 1× with sterile RNase free PBS. Cell Extraction was carried out using ice-cold solutions as follows: Slides were incubated for 30 sec in CSK buffer (100 mM NaCl/300 mM sucrose/3 mM MgCl_2_/10 mM PIPES, pH 6.8), 10 min in CSK buffer/0.1% Triton X-100, followed by 30 sec in CSK buffer. Cells were then fixed in 4% paraformaldehyde in PBS for 10 min and stored in 70% EtOH at −20°C until use. Just prior to RNA FISH, slides were dehydrated through an EtOH series and allowed to air dry. Denatured probes were prehybridized at 37°C for 10 min, applied to nondenatured slides and hybridized at 37°C for 14–16 h. Post-hybridization washes consisted of one 3-min wash in 50% formamide/2× SSC at 40°C followed by one 2-min rinse in 2× SSC/0.1% TX-100 for 1 min at RT. Slides were then fixed in 4% paraformaldehyde in PBS for 5 min at RT, and briefly rinsed in 2× SSC/0.1% TX-100 at RT. Coverslips were mounted with Prolong Gold antifade plus DAPI (Invitrogen) and slides were viewed under UV fluorescence (Olympus). Z-stack images were generated using a Cytovision workstation. After capturing RNA FISH signals, the coverslips were removed, the slides were dehydrated in an ethanol series, and then processed for DNA FISH, beginning with the RNase treatment step, as described above.

### Replication timing assay

The BrdU replication timing assay was performed as described previously on exponentially dividing cultures and asynchronously growing cells (Smith and Thayer 2012). Mitotic chromosome spreads were prepared, and DNA FISH was performed as described above. The incorporated BrdU was then detected using a FITC-labeled anti-BrdU antibody (Roche). Coverslips were mounted with Prolong Gold antifade plus DAPI (Invitrogen), and viewed under UV fluorescence. All images were captured with an Olympus BX Fluorescent Microscope using a 100× objective, automatic filter-wheel and Cytovision workstation. Individual chromosomes were identified with either chromosome-specific paints, centromeric probes, BACs or by inverted DAPI staining. Utilizing the Cytovision workstation, each chromosome was isolated from the metaphase spread and a line drawn along the middle of the entire length of the chromosome. The Cytovision software was used to calculate the pixel area and intensity along each chromosome for each fluorochrome occupied by the DAPI and BrdU (FITC) signals. The total amount of fluorescent signal in each chromosome was calculated by multiplying the average pixel intensity by the area occupied by those pixels. The BrdU incorporation into human chromosome 6 homologs containing CRISPR/Cas9 modifications was calculated by dividing the total incorporation into the chromosome with the smaller chromosome 6 centromere (6B) divided by the BrdU incorporation into the chromosome 6 with the larger centromere (6A) within the same cell. Boxplots were generated from data collected from eight to 12 cells per clone or treatment group. Differences in measurements were tested across categorical groupings by using the Kruskal–Wallis test (Kruskal 1964) and listed as *P*-values for the corresponding plots.

### CRISPR/Cas9 engineering

Using Lipofectamine 2000, according to the manufacturer's recommendations, we cotransfected HTD114 cells with plasmids encoding GFP, sgRNAs, and Cas9 endonuclease (Origene). Each plasmid-encoded sgRNAs were designed to bind at the indicated locations (Fig. 1; also see Supplemental Table S1). Forty-eight hours after transfection, cells were plated at clonal density and allowed to expand for 2–3 wk. The presence of deletions was confirmed by PCR using the primers described in Supplemental Table S1. The single-cell colonies that grew were analyzed for heterozygous deletions by PCR. We used retention of a heterozygous SNPs (see Supplemental Table S1) to identify the disrupted allele (CHR6A vs. CHR6B), and homozygosity at this SNP confirmed that cell clones were homogenous.

## SUPPLEMENTAL MATERIAL

Supplemental material is available for this article.

## Supplementary Material

Supplemental Material

## References

[RNA073114HESC1] Alexander MK, Mlynarczyk-Evans S, Royce-Tolland M, Plocik A, Kalantry S, Magnuson T, Panning B. 2007 Differences between homologous alleles of olfactory receptor genes require the Polycomb Group protein Eed. J Cell Biol 179: 269–276. 10.1083/jcb.200706053 17954609 PMC2064763

[RNA073114HESC2] Allen E, Horvath S, Tong F, Kraft P, Spiteri E, Riggs AD, Marahrens Y. 2003 High concentrations of long interspersed nuclear element sequence distinguish monoallelically expressed genes. Proc Natl Acad Sci 100: 9940–9945. 10.1073/pnas.1737401100 12909712 PMC187893

[RNA073114HESC3] Attig J, Agostini F, Gooding C, Chakrabarti AM, Singh A, Haberman N, Zagalak JA, Emmett W, Smith CWJ, Luscombe NM, 2018 Heteromeric RNP assembly at LINEs controls lineage-specific RNA processing. Cell 174: 1067–1081.e17. 10.1016/j.cell.2018.07.001 30078707 PMC6108849

[RNA073114HESC4] Bailey JA, Carrel L, Chakravarti A, Eichler EE. 2000 Molecular evidence for a relationship between LINE-1 elements and X chromosome inactivation: the Lyon repeat hypothesis. Proc Natl Acad Sci 97: 6634–6639. 10.1073/pnas.97.12.6634 10841562 PMC18684

[RNA073114HESC5] Bartholdy B, Mukhopadhyay R, Lajugie J, Aladjem MI, Bouhassira EE. 2015 Allele-specific analysis of DNA replication origins in mammalian cells. Nat Commun 6: 7051 10.1038/ncomms8051 25987481 PMC4479011

[RNA073114HESC6] Bartolomei MS. 2009 Genomic imprinting: employing and avoiding epigenetic processes. Genes Dev 23: 2124–2133. 10.1101/gad.1841409 19759261 PMC2751984

[RNA073114HESC7] Breger KS, Smith L, Turker MS, Thayer MJ. 2004 Ionizing radiation induces frequent translocations with delayed replication and condensation. Cancer Res 64: 8231–8238. 10.1158/0008-5472.CAN-04-0879 15548689

[RNA073114HESC8] Breger KS, Smith L, Thayer MJ. 2005 Engineering translocations with delayed replication: evidence for *cis* control of chromosome replication timing. Hum Mol Genet 14: 2813–2827. 10.1093/hmg/ddi314 16115817

[RNA073114HESC9] Bryois J, Buil A, Evans DM, Kemp JP, Montgomery SB, Conrad DF, Ho KM, Ring S, Hurles M, Deloukas P, 2014 *Cis* and *trans* effects of human genomic variants on gene expression. PLoS Genet 10: e1004461 10.1371/journal.pgen.1004461 25010687 PMC4091791

[RNA073114HESC10] Caron M, St-Onge P, Drouin S, Richer C, Sontag T, Busche S, Bourque G, Pastinen T, Sinnett D. 2018 Very long intergenic non-coding RNA transcripts and expression profiles are associated to specific childhood acute lymphoblastic leukemia subtypes. PLoS One 13: e0207250 10.1371/journal.pone.0207250 30440012 PMC6237371

[RNA073114HESC11] Chang BH, Smith L, Huang J, Thayer M. 2007 Chromosomes with delayed replication timing lead to checkpoint activation, delayed recruitment of Aurora B and chromosome instability. Oncogene 26: 1852–1861. 10.1038/sj.onc.1209995 17001311 PMC3285441

[RNA073114HESC12] Chen J, Rattner A, Nathans J. 2006 Effects of L1 retrotransposon insertion on transcript processing, localization and accumulation: lessons from the retinal degeneration 7 mouse and implications for the genomic ecology of L1 elements. Hum Mol Genet 15: 2146–2156. 10.1093/hmg/ddl138 16723373

[RNA073114HESC13] Chess A. 2012 Mechanisms and consequences of widespread random monoallelic expression. Nat Rev Genet 13: 421–428. 10.1038/nrg3239 22585065

[RNA073114HESC14] Dileep V, Gilbert DM. 2018 Single-cell replication profiling to measure stochastic variation in mammalian replication timing. Nat Commun 9: 427 10.1038/s41467-017-02800-w 29382831 PMC5789892

[RNA073114HESC15] Dobin A, Davis CA, Schlesinger F, Drenkow J, Zaleski C, Jha S, Batut P, Chaisson M, Gingeras TR. 2013 STAR: ultrafast universal RNA-seq aligner. Bioinformatics 29: 15–21. 10.1093/bioinformatics/bts635 23104886 PMC3530905

[RNA073114HESC16] Donley N, Stoffregen EP, Smith L, Montagna C, Thayer MJ. 2013 Asynchronous replication, mono-allelic expression, and long range *cis*-effects of ASAR6. PLoS Genet 9: e1003423 10.1371/journal.pgen.1003423 23593023 PMC3617217

[RNA073114HESC17] Donley N, Smith L, Thayer MJ. 2015 ASAR15, a *cis*-acting locus that controls chromosome-wide replication timing and stability of human chromosome 15. PLoS Genet 11: e1004923 10.1371/journal.pgen.1004923 25569254 PMC4287527

[RNA073114HESC18] Ensminger AW, Chess A. 2004 Coordinated replication timing of monoallelically expressed genes along human autosomes. Hum Mol Genet 13: 651–658. 10.1093/hmg/ddh062 14734625

[RNA073114HESC19] Fragkos M, Ganier O, Coulombe P, Mechali M. 2015 DNA replication origin activation in space and time. Nat Rev Mol Cell Biol 16: 360–374. 10.1038/nrm4002 25999062

[RNA073114HESC20] Galupa R, Heard E. 2018 X-chromosome inactivation: a crossroads between chromosome architecture and gene regulation. Annu Rev Genet 52: 535–566. 10.1146/annurev-genet-120116-024611 30256677

[RNA073114HESC21] Gendrel A-V, Attia M, Chen C-J, Diabangouaya P, Servant N, Barillot E, Heard E. 2014 Developmental dynamics and disease potential of random monoallelic gene expression. Dev Cell 28: 366–380. 10.1016/j.devcel.2014.01.016 24576422

[RNA073114HESC22] Gendrel A-V, Marion-Poll L, Katoh K, Heard E. 2016 Random monoallelic expression of genes on autosomes: parallels with X-chromosome inactivation. Semin Cell Dev Biol 56: 100–110. 10.1016/j.semcdb.2016.04.007 27101886

[RNA073114HESC23] Gimelbrant A, Hutchinson JN, Thompson BR, Chess A. 2007 Widespread monoallelic expression on human autosomes. Science 318: 1136–1140. 10.1126/science.1148910 18006746

[RNA073114HESC24] Goldmit M, Bergman Y. 2004 Monoallelic gene expression: a repertoire of recurrent themes. Immunol Rev 200: 197–214. 10.1111/j.0105-2896.2004.00158.x 15242406

[RNA073114HESC25] Hiratani I, Leskovar A, Gilbert DM. 2004 Differentiation-induced replication-timing changes are restricted to AT-rich/long interspersed nuclear element (LINE)-rich isochores. Proc Natl Acad Sci 101: 16861–16866. 10.1073/pnas.0406687101 15557005 PMC534734

[RNA073114HESC26] Kapranov P, St Laurent G, Raz T, Ozsolak F, Reynolds CP, Sorensen PH, Reaman G, Milos P, Arceci RJ, Thompson JF, 2010 The majority of total nuclear-encoded non-ribosomal RNA in a human cell is ‘dark matter’ un-annotated RNA. BMC Biol. 8: 149 10.1186/1741-7007-8-149 21176148 PMC3022773

[RNA073114HESC27] Kruskal JB. 1964 Multidimensional scaling by optimizing goodness of fit to a nonmetric hypothesis. Psychometrika 29: 1–27. 10.1007/BF02289565

[RNA073114HESC28] Lehman A, Thouta S, Mancini GMS, Naidu S, van Slegtenhorst M, McWalter K, Person R, Mwenifumbo J, Salvarinova R, Guella I, 2017 Loss-of-function and gain-of-function mutations in KCNQ5 cause intellectual disability or epileptic encephalopathy. Am J Hum Genet 101: 65–74. 10.1016/j.ajhg.2017.05.016 28669405 PMC5501867

[RNA073114HESC29] Li H, Handsaker B, Wysoker A, Fennell T, Ruan J, Homer N, Marth G, Abecasis G, Durbin R. 2009 The sequence alignment/map format and SAMtools. Bioinformatics 25: 2078–2079. 10.1093/bioinformatics/btp352 19505943 PMC2723002

[RNA073114HESC30] Li SM, Valo Z, Wang J, Gao H, Bowers CW, Singer-Sam J. 2012 Transcriptome-wide survey of mouse CNS-derived cells reveals monoallelic expression within novel gene families. PLoS One 7: e31751 10.1371/journal.pone.0031751 22384067 PMC3285176

[RNA073114HESC31] Lin M, Hrabovsky A, Pedrosa E, Wang T, Zheng D, Lachman HM. 2012 Allele-biased expression in differentiating human neurons: implications for neuropsychiatric disorders. PLoS One 7: e44017 10.1371/journal.pone.0044017 22952857 PMC3431331

[RNA073114HESC32] Luedi PP, Dietrich FS, Weidman JR, Bosko JM, Jirtle RL, Hartemink AJ. 2007 Computational and experimental identification of novel human imprinted genes. Genome Res 17: 1723–1730. 10.1101/gr.6584707 18055845 PMC2099581

[RNA073114HESC33] Lyon MF. 1998 X-chromosome inactivation: a repeat hypothesis. Cytogenet Cell Genet 80: 133–137. 10.1159/000014969 9678347

[RNA073114HESC34] Lyon MF. 2003 The Lyon and the LINE hypothesis. Semin Cell Dev Biol 14: 313–318. 10.1016/j.semcdb.2003.09.015 15015738

[RNA073114HESC35] Mostoslavsky R, Singh N, Tenzen T, Goldmit M, Gabay C, Elizur S, Qi P, Reubinoff BE, Chess A, Cedar H, 2001 Asynchronous replication and allelic exclusion in the immune system. Nature 414: 221–225. 10.1038/35102606 11700561

[RNA073114HESC36] Petretto E, Mangion J, Dickens NJ, Cook SA, Kumaran MK, Lu H, Fischer J, Maatz H, Kren V, Pravenec M, 2006 Heritability and tissue specificity of expression quantitative trait loci. PLoS Genet 2: e172 10.1371/journal.pgen.0020172 17054398 PMC1617131

[RNA073114HESC37] Platt EJ, Smith L, Thayer MJ. 2018 L1 retrotransposon antisense RNA within ASAR lncRNAs controls chromosome-wide replication timing. J Cell Biol 217: 541–553. 10.1083/jcb.201707082 29288153 PMC5800813

[RNA073114HESC38] Schlesinger S, Selig S, Bergman Y, Cedar H. 2009 Allelic inactivation of rDNA loci. Genes Dev 23: 2437–2447. 10.1101/gad.544509 19833769 PMC2764490

[RNA073114HESC39] Signor SA, Nuzhdin SV. 2018 The evolution of gene expression in *cis* and *trans*. Trends Genet 34: 532–544. 10.1016/j.tig.2018.03.007 29680748 PMC6094946

[RNA073114HESC40] Singh N, Ebrahimi FA, Gimelbrant AA, Ensminger AW, Tackett MR, Qi P, Gribnau J, Chess A. 2003 Coordination of the random asynchronous replication of autosomal loci. Nat Genet 33: 339–341. 10.1038/ng1102 12577058

[RNA073114HESC41] Smith L, Thayer M. 2012 Chromosome replicating timing combined with fluorescent in situ hybridization. J Vis Exp 10: e4400 10.3791/4400 PMC3567166 23271586

[RNA073114HESC42] Smith L, Plug A, Thayer M. 2001 Delayed replication timing leads to delayed mitotic chromosome condensation and chromosomal instability of chromosome translocations. Proc Natl Acad Sci 98: 13300–13305. 10.1073/pnas.241355098 11698686 PMC60865

[RNA073114HESC43] St Laurent G, Savva YA, Kapranov P. 2012 Dark matter RNA: an intelligent scaffold for the dynamic regulation of the nuclear information landscape. Front Genet 3: 57 10.3389/fgene.2012.00057 22539933 PMC3336093

[RNA073114HESC44] St Laurent G, Shtokalo D, Dong B, Tackett MR, Fan X, Lazorthes S, Nicolas E, Sang N, Triche TJ, McCaffrey TA, 2013 VlincRNAs controlled by retroviral elements are a hallmark of pluripotency and cancer. Genome Biol 14: R73 10.1186/gb-2013-14-7-r73 23876380 PMC4053963

[RNA073114HESC45] St Laurent G, Vyatkin Y, Antonets D, Ri M, Qi Y, Saik O, Shtokalo D, de Hoon MJ, Kawaji H, Itoh M, 2016 Functional annotation of the vlinc class of non-coding RNAs using systems biology approach. Nucleic Acids Res 44: 3233–3252. 10.1093/nar/gkw162 27001520 PMC4838384

[RNA073114HESC46] Stoffregen EP, Donley N, Stauffer D, Smith L, Thayer MJ. 2011 An autosomal locus that controls chromosome-wide replication timing and mono-allelic expression. Hum Mol Genet 20: 2366–2378. 10.1093/hmg/ddr138 21459774 PMC3098730

[RNA073114HESC47] Thayer MJ. 2012 Mammalian chromosomes contain *cis*-acting elements that control replication timing, mitotic condensation, and stability of entire chromosomes. Bioessays 34: 760–770. 10.1002/bies.201200035 22706734 PMC3517107

[RNA073114HESC48] Zhu Y, Bye S, Stambrook PJ, Tischfield JA. 1994 Single-base deletion induced by benzo[*a*]pyrene diol epoxide at the adenine phosphoribosyltransferase locus in human fibrosarcoma cell lines. Mutat Res 321: 73–79. 10.1016/0165-1218(94)90122-8 7510848

