# Supplementary material for: Reciprocal monoallelic expression of ASAR lncRNA genes controls replication timing of human chromosome 6
Source: RNA. 2020 Jun;26(6):724–38. doi: 10.1261/rna.073114.119 (PMC7266157; doi:10.1261/rna.073114.119)
Supplement: Supplemental Material [file supp_26_6_724__index.html]

Reciprocal monoallelic expression of ASAR lncRNA genes controls replication timing of human chromosome 6 — Supplemental Material 

# Reciprocal monoallelic expression of ASAR lncRNA genes controls replication timing of human chromosome 6

## Supplemental Material

- Supplemental\_Fig\_S1.tif
- Supplemental\_Fig\_S2.tif
- Supplemental\_Fig\_S3.tif
- Supplemental\_Fig\_S4.tif
- Supplemental\_Fig\_S5.tif
- Supplemental\_Fig\_S6.tif
- Supplemental\_Fig\_S7.tif
- Supplemental\_Table\_S1.xlsx
- Supplemental\_Table\_S2.xlsx
- Supplemental\_Table\_S3.xlsx
- Supplemental\_Table\_S4.xlsx
- Supplemental\_Legends.docx
