# Supplementary figures and images for: Reciprocal monoallelic expression of ASAR lncRNA genes controls replication timing of human chromosome 6
Source: RNA. 2020 Jun;26(6):724–38. doi: 10.1261/rna.073114.119 (PMC7266157; doi:10.1261/rna.073114.119)

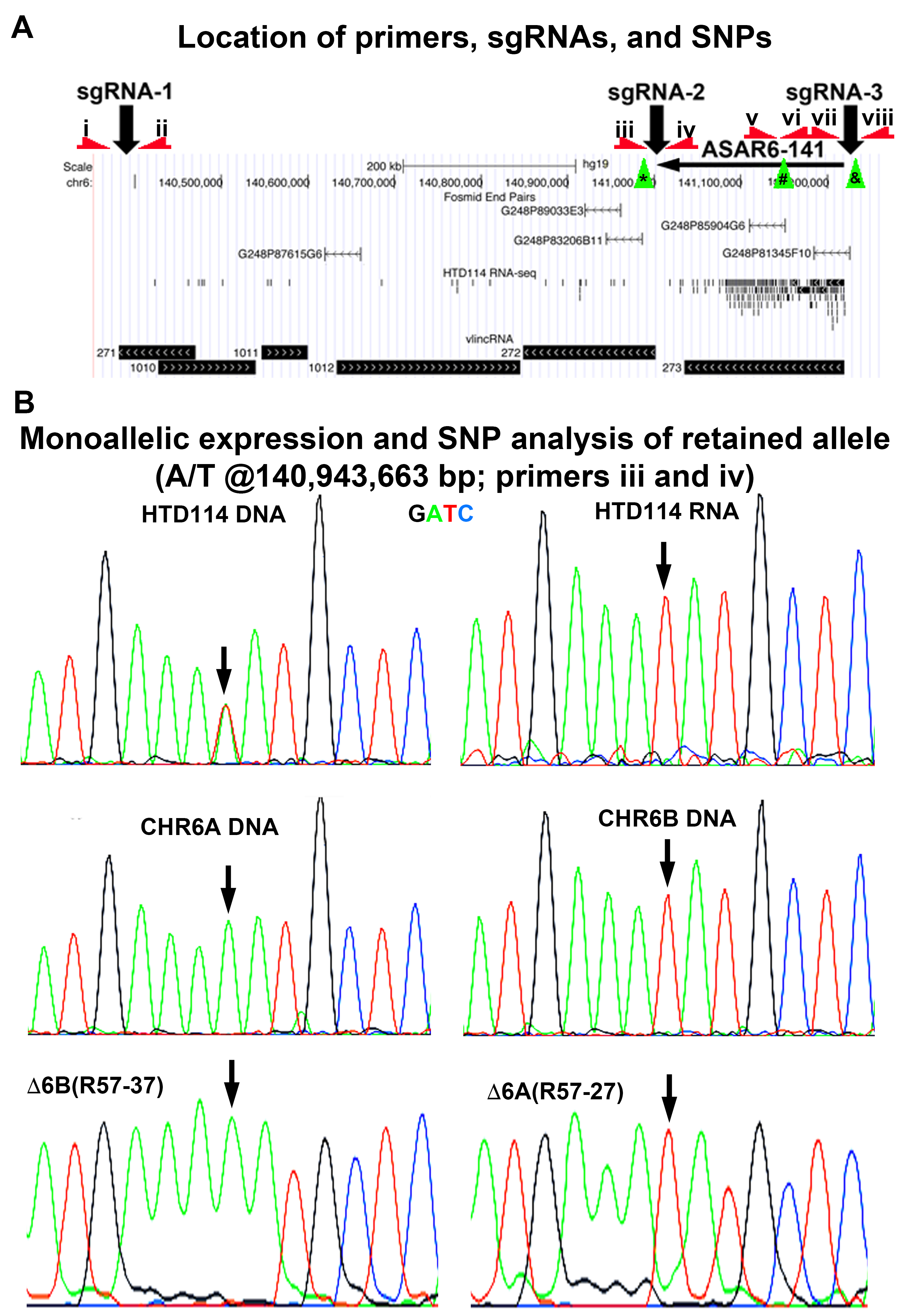

Supplement: Supplemental Material [file supp_073114.119_Supplemental_Fig_S1.tif]

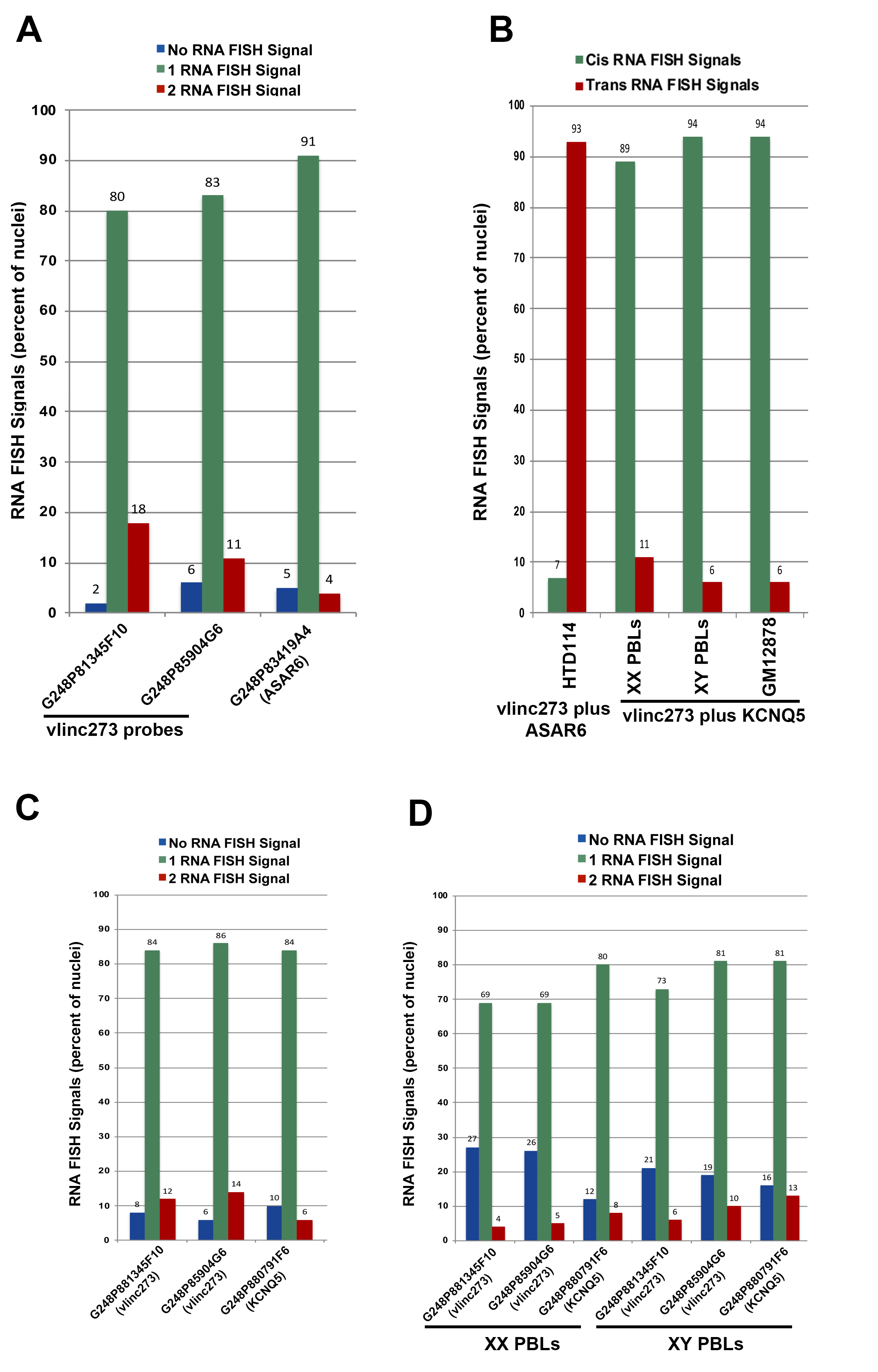

Supplement: Supplemental Material [file supp_073114.119_Supplemental_Fig_S2.tif]

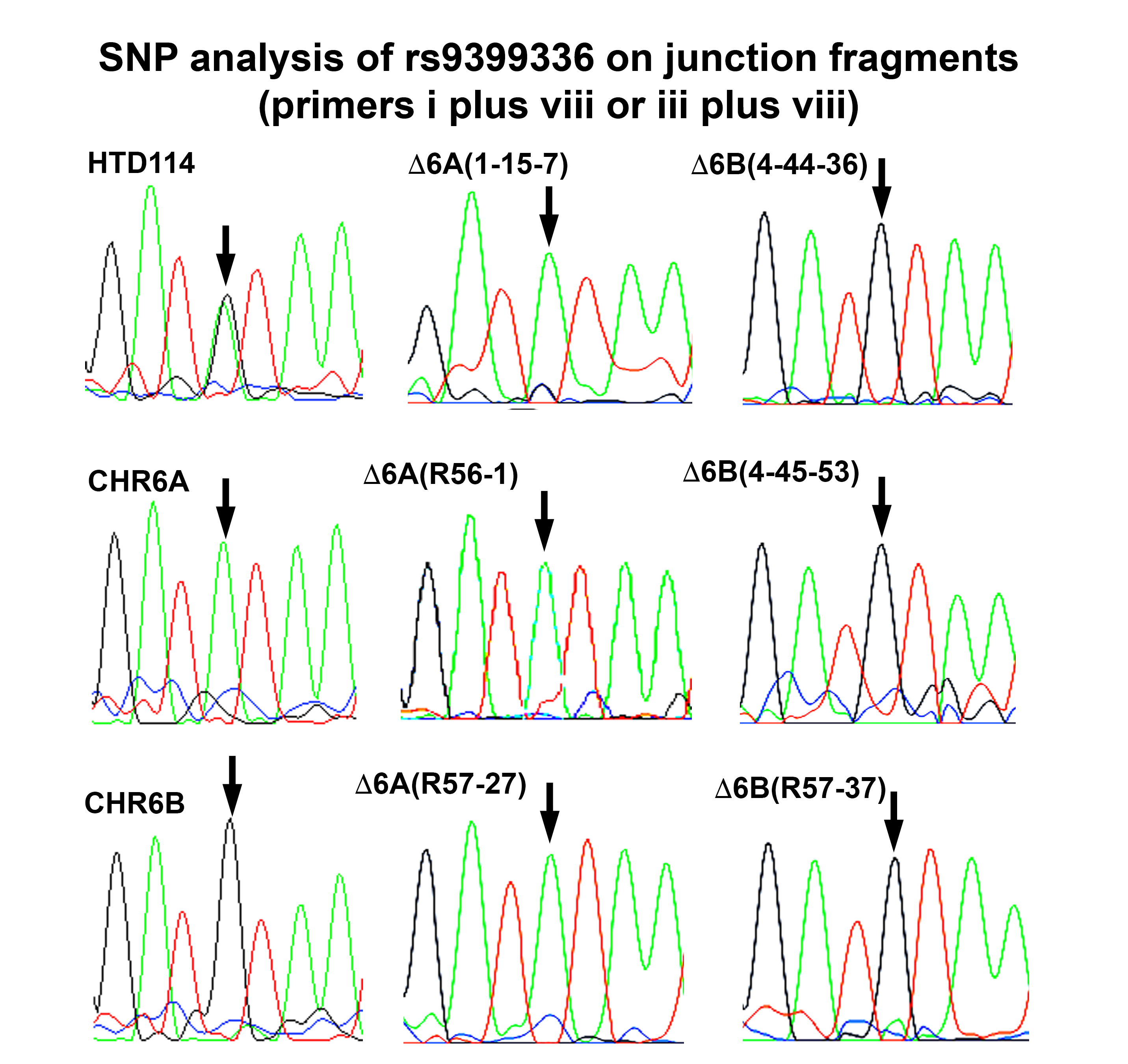

Supplement: Supplemental Material [file supp_073114.119_Supplemental_Fig_S3.tif]

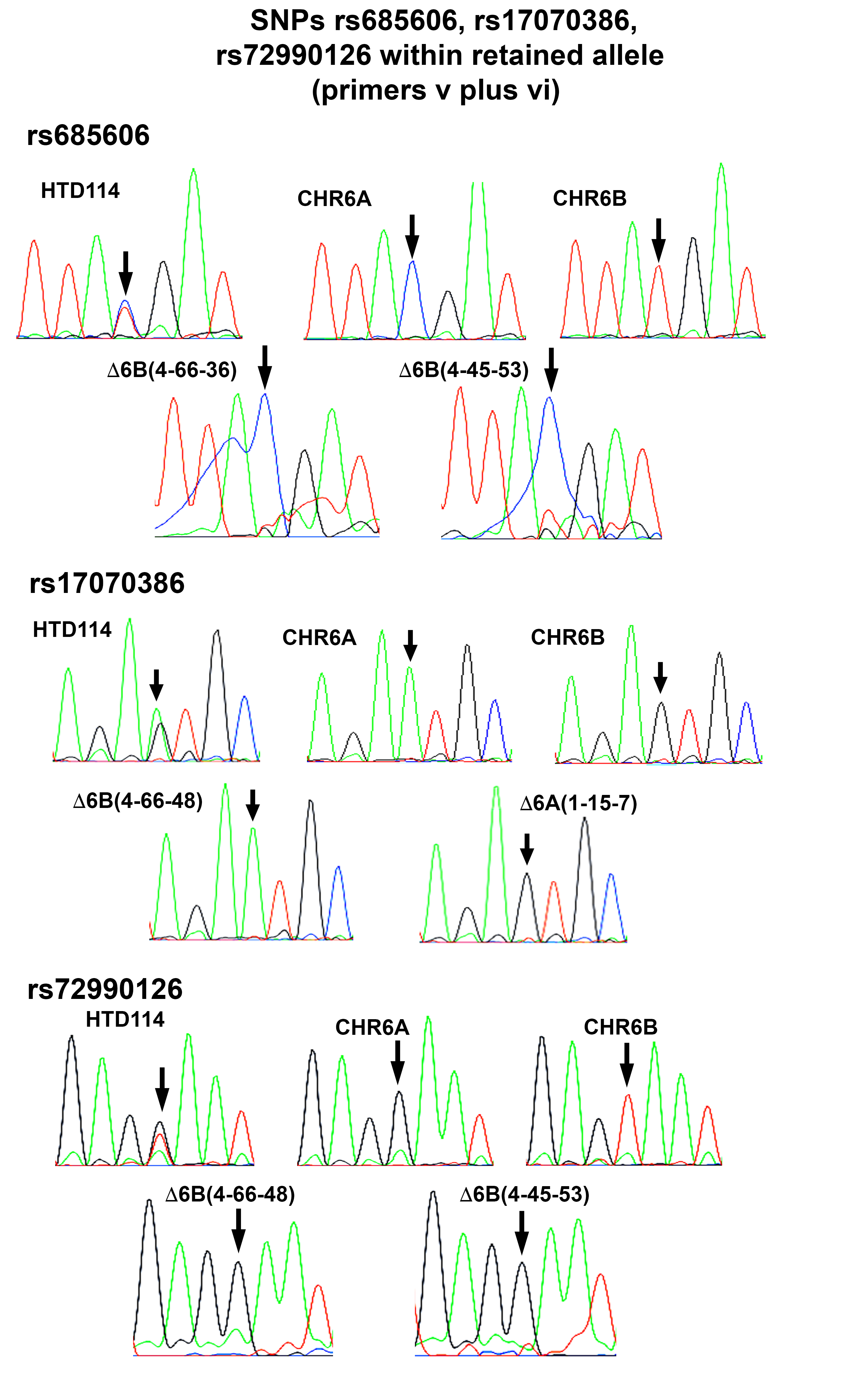

Supplement: Supplemental Material [file supp_073114.119_Supplemental_Fig_S4.tif]

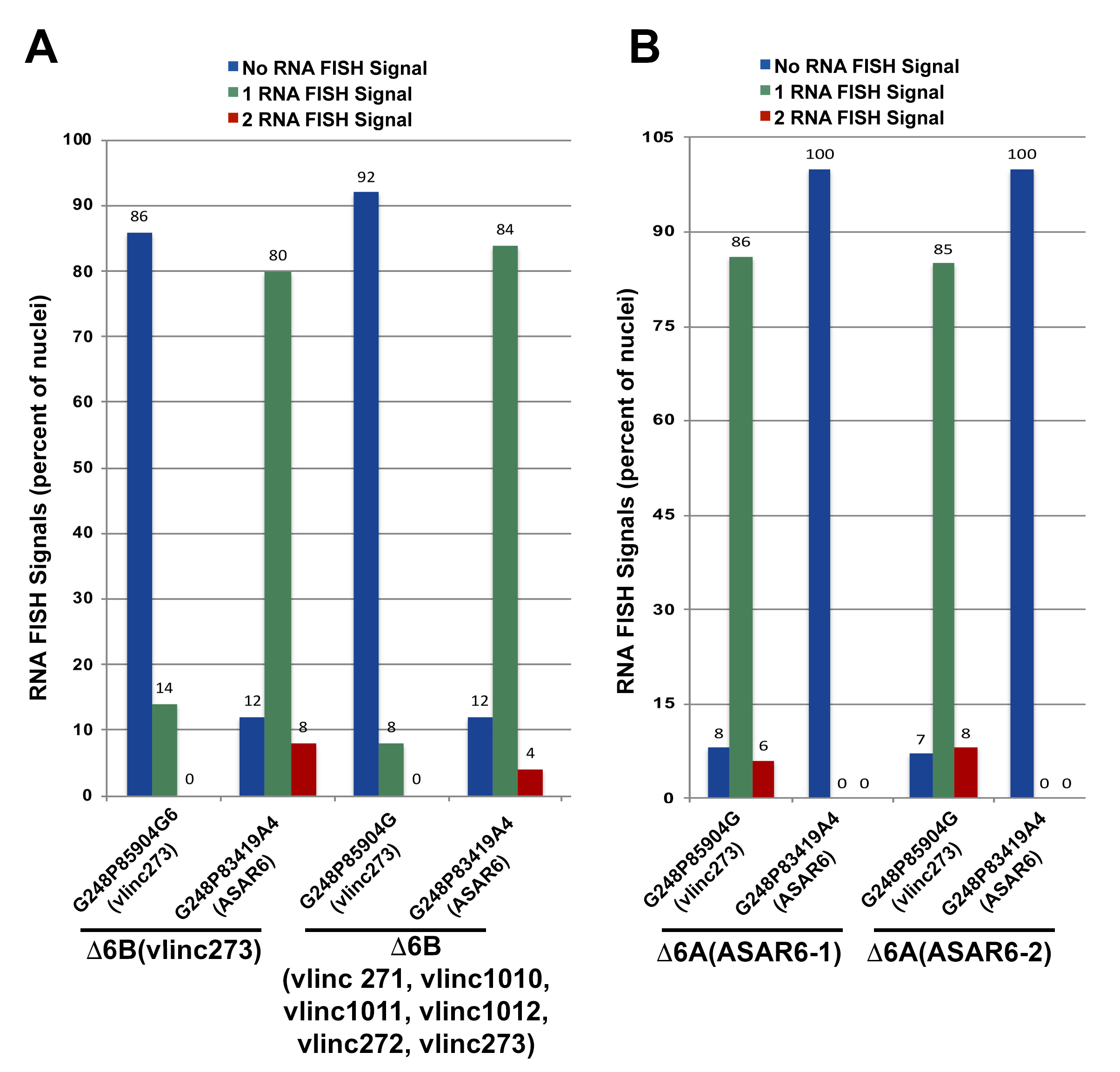

Supplement: Supplemental Material [file supp_073114.119_Supplemental_Fig_S5.tif]

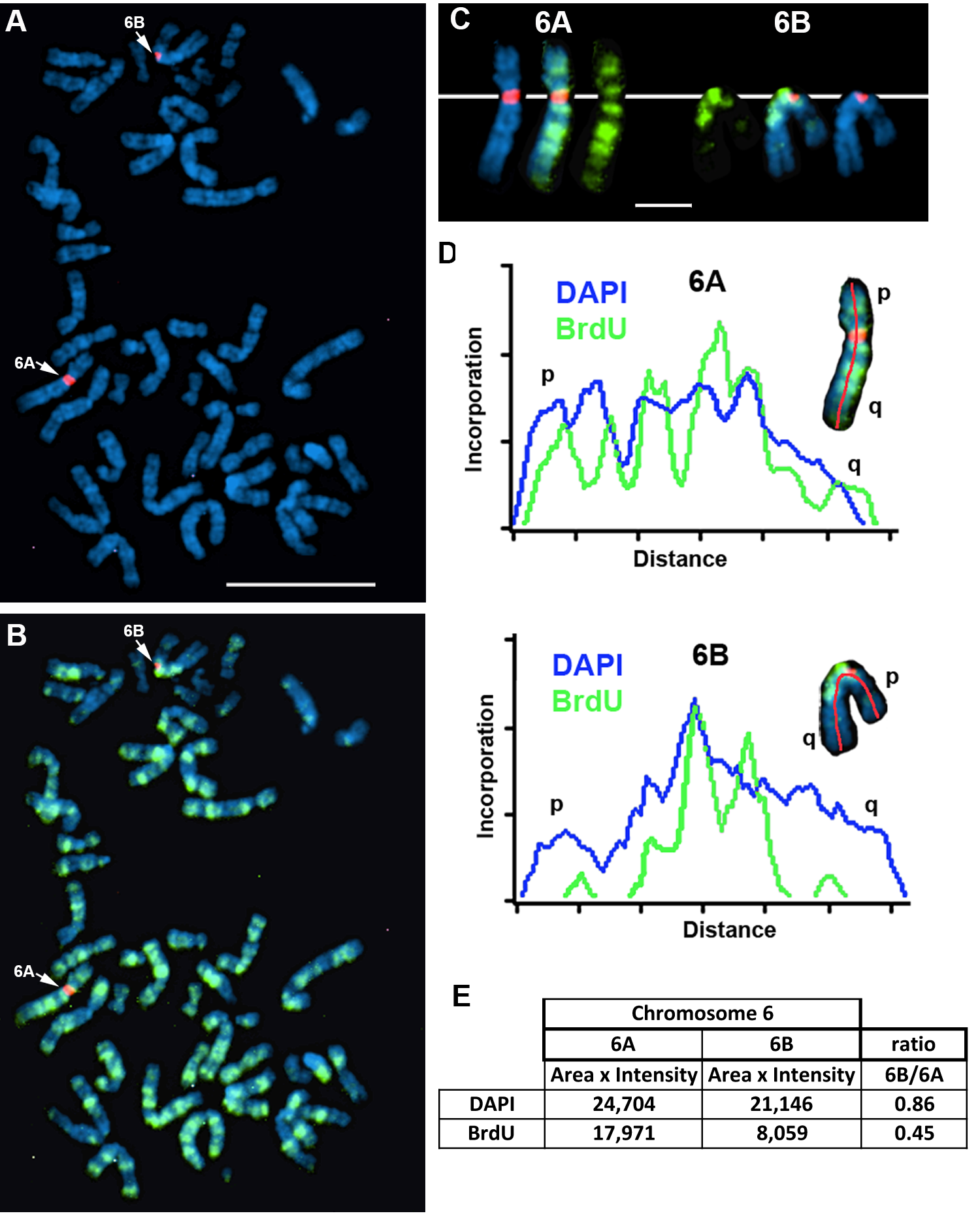

Supplement: Supplemental Material [file supp_073114.119_Supplemental_Fig_S6.tif]

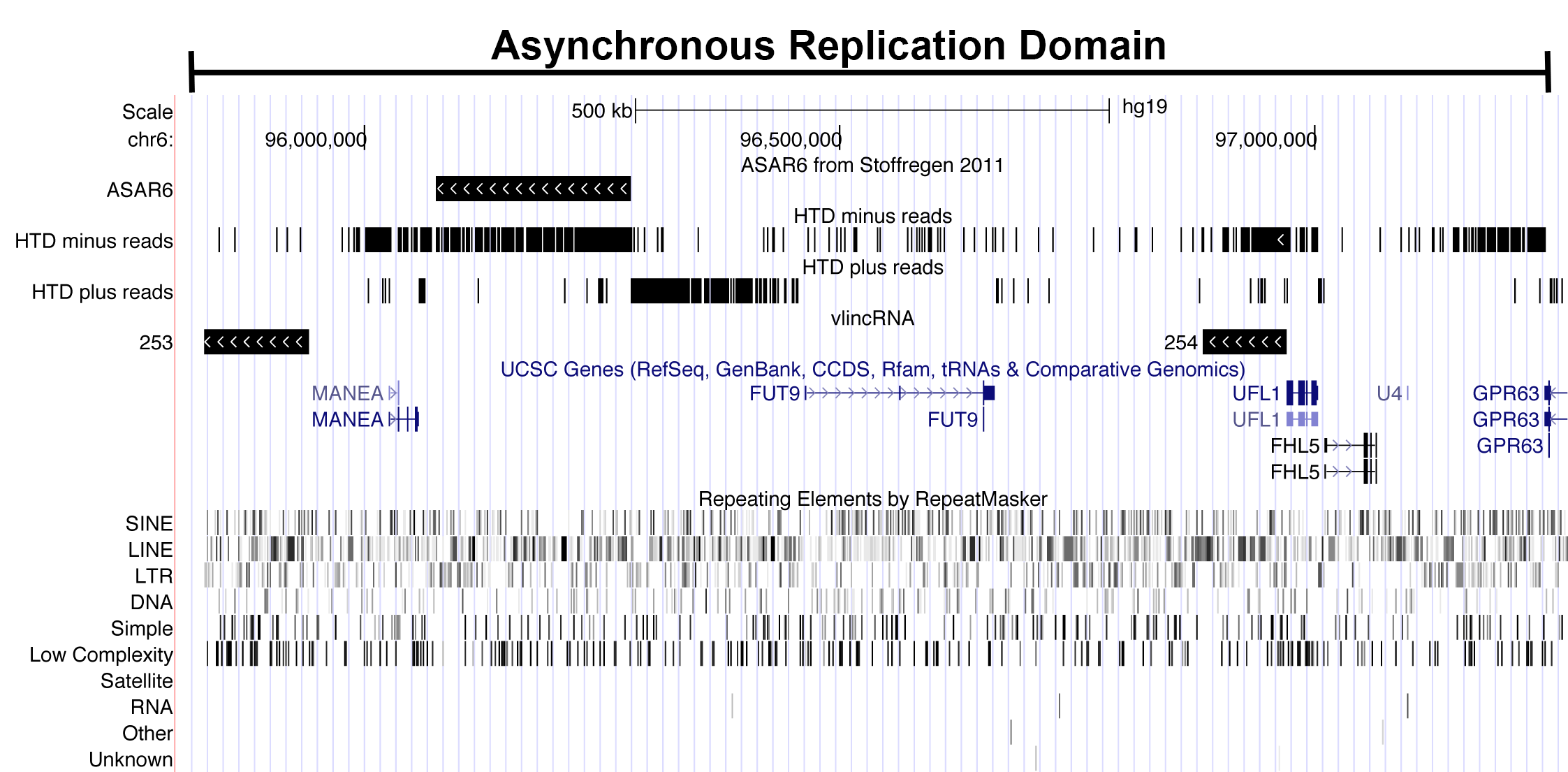

Supplement: Supplemental Material [file supp_073114.119_Supplemental_Fig_S7.tif]
