# Supplementary material for: Reciprocal monoallelic expression of ASAR lncRNA genes controls replication timing of human chromosome 6
Source: RNA. 2020 Jun;26(6):724–38. doi: 10.1261/rna.073114.119 (PMC7266157; doi:10.1261/rna.073114.119)
Supplement: Supplemental Material [file supp_073114.119_Supplemental_Legends.docx]

**Supporting Information**

**Figure S1**. Monoallelic expression and heterozygous deletions of the vlinc cluster at ~141 mb of chromosome 6. (A) UCSC Genome Browser view of the vlinc cluster on chromosome 6 between 140.3 and 141.3 mb showing the location of the vlincRNA genes (vlinc271, vlinc1010, vlinc1011, vlinc1012, vlinc272, and vlinc273), Fosmids, PCR primers (red half arrows marked i-viii; see Table S2), sgRNAs (see Table S2), and heterozygous SNPs (see Table S2). Green triangles show the location of the heterozygous SNPs corresponding to: (*) rs2328092, rs989613623, and A/T at 140,943,663, (#) rs685606, rs17070386, rs72990126, and (&) rs9399336. (B) DNA sequencing traces from PCR products (using primers iii and iv; see Table S2), which show the A/T SNP at 140,943,663. PCRs were carried out on genomic DNAs isolated from HTD114, two mono-chromosomal hybrids containing the two different chromosome 6s from HTD114 {L(Hyg)-1 contains chromosome 6A (CHR6A) and expresses *ASAR6*, and L(Neo)-38 contains chromosome 6B (CHR6B) and is silent for *ASAR6* (Stoffregen et al. 2011)}. The sequencing trace from HTD114 cDNA (RNA) is shown. The bottom two panels, marked Δ6B(R57-37) and Δ6A(R57-27), show the sequencing traces from PCR products produced from genomic DNA isolated from heterozygous deletions of the entire vlinc cluster (using sgRNA-1 and sgRNA-3) from CHR6B and CHR6A, respectively. Note that these PCR products are generated from the non-deleted alleles. The arrows mark the location of the heterozygous SNP.

**Figure S2.** Quantitation of RNA FISH signals. (A) RNA-DNA FISH to detect vlinc273 and ASAR6 expression in HTD114 cells. Fosmids G248P81345F10 and G248P85904G6 were used as probe to detect vlinc273 RNA and Fosmid G248P86031A6 was used as probe to detect ASAR6 RNA. RNA FISH signals were scored in >200 cells and the percent of cells with 1 (green), 2 (red), or No (blue) signals are shown. (B) RNA-DNA FISH was used to detect vlinc273 (G248P81345F10 probe) plus ASAR6 (G248P86031A6 probe) RNAs simultaneously in HTD114 cells. A chromosome 6 whole chromosome paint or chromosome 6 centromeric probe was used to detect chromosome 6 DNA. RNA-DNA FISH was used to detect vlinc273 (G248P81345F10 probe) plus *KCNQ5* (G248P880791F6) expression in primary blood lymphocytes isolated from 2 unrelated individuals, one female (XX) and one male (XY), and in GM12878 cells. The percentage of nuclei showing hybridization to the same (i.e. in *cis*) or opposite (i.e. in *trans*) chromosome 6s. A minimum of 100 cells with both signals were scored for each assay, and the percentage of nuclei showing hybridization to the same (i.e. in *cis*; green) or opposite (i.e. in *trans*; red) chromosome 6s is shown. (C) RNA-DNA FISH was used to detect vlinc273 (G248P81345F10 probe) plus *KCNQ5* (G248P880791F6 probe) RNAs in GM12878 cells. A chromosome 6 centromeric probe was used to detect chromosome 6 DNA. RNA FISH signals were scored in >200 cells and the percent of cells with 1 (green), 2 (red), or No (blue) signals are shown. (D) RNA-DNA FISH was used to detect vlinc273 (G248P81345F10 probe) plus *KCNQ5* (G248P880791F6 probe) RNAs simultaneously in primary blood lymphocytes isolated from 2 unrelated individuals, one female (XX) and one male (XY). A chromosome 6 centromeric probe was used to detect chromosome 6 DNA. RNA FISH signals were scored in >200 cells and the percent of cells with 1 (green), 2 (red), or No (blue) signals are shown.

**Figure S3**. DNA sequencing traces from PCR products generated using either primers vii plus viii (HTD114, CHR6A, and CHR6B), iii plus viii {Δ6A(1-15-7), Δ6B(4-44-36), Δ6B(4-45-53); see Fig. S1A and Table S2} or i plus viii {Δ6A(R56-1), Δ6A(R57-27), Δ6B(R57-37)}. All of the sequence traces show the DNA region containing the heterozygous SNP rs9399336 (see Table S2), which is marked by arrows. PCR products were generated using primers vii plus viii on genomic DNA isolated from HTD114 and two mono-chromosomal hybrids containing the two different chromosome 6s from HTD114 {L(Hyg)-1 contains chromosome 6A (CHR6A), and L(Neo)-38 contains chromosome 6B (CHR6B) (Stoffregen et al. 2011)}. In addition, sequence traces from 6 independent CRISPR/Cas9-mediated deletions, containing deletions of either vlinc273 alone {Δ6A(1-15-7), Δ6B(4-44-36), Δ6B(4-45-53)} or deletions of the entire vlincRNA gene cluster {Δ6A(R56-1), Δ6A(R57-27), Δ6B(R57-37). Note that these primer combinations amplify junction fragments generated when only vlinc273 is deleted (primers iii plus viii), or when the entire vlinc cluster is deleted (primers i plus viii). Therefore, these junction PCR products are generated from the deleted chromosome, i.e. either 6A or 6B.

**Figure S4.** Examples of DNA sequencing traces from PCR products generated using primers v plus vii (see Fig. S1A and Table S2). The sequence traces show the DNA regions containing the heterozygous SNPs rs685606, rs17070386, and rs72990126 (see Table S2) The location of the SNPs are marked by arrows. PCR products were generated from genomic DNA isolated from HTD114 and the two mono-chromosomal hybrids containing the two different chromosome 6s from HTD114 {L(Hyg)-1 contains chromosome 6A (CHR6A), and L(Neo)-38 contains chromosome 6B (CHR6B) (Stoffregen et al. 2011)}. In addition, sequence traces from 4 independent CRISPR/Cas9-mediated deletions, containing deletions of vlinc273 {Δ6A(1-15-7), Δ6B(4-66-36), Δ6B(4-66-48), Δ6B(4-45-53)}. Note that these PCR products are generated from the non-deleted allele. The arrows mark the locations of the heterozygous SNPs.

**Figure S5.** Heterozygous deletions affect the expressed alleles of vlinc273 or ASAR6. (A) RNA-DNA FISH was used to detect vlinc273 and ASAR6 expression in cells with heterozygous deletions of the expressed alleles of vlinc273 {Δ6B(vlinc273)} or the entire vlinc RNA cluster {Δ6B(vlinc271, vlinc1010, vlinc1011, vlinc1012, vlinc272, vlinc273)}. Fosmid G248P81345F10 was used as probe to detect vlinc273 RNA and Fosmid G248P86031A6 was used as probe to detect ASAR6 RNA. RNA FISH signals were scored in >200 cells and the percent of cells with 1 (green), 2 (red), or No (blue) signals are shown. (B) RNA-DNA FISH was used to detect vlinc273 and ASAR6 expression in cells with heterozygous deletions of the expressed alleles of *ASAR6* {Δ6A(ASAR6-1) and Δ6A(ASAR6-2)}. Fosmid G248P81345F10 was used as probe to detect vlinc273 RNA and Fosmid G248P86031A6 was used as probe to detect ASAR6 RNA. RNA FISH signals were scored in >200 cells and the percent of cells with 1 (green), 2 (red), or No (blue) signals are shown.

**Figure S6.** Delayed replication of chromosome 6 following disruption of *ASAR6*. **(**A and B) A representative mitotic spread from BrdU (green) treated cells containing a deletion of the expressed allele of *ASAR6* (Platt et al. 2017). Mitotic cells were subjected to DNA FISH using a chromosome 6 centromeric probe (red). The larger centromere resides on the chromosome 6 with the expressed *ASAR6* allele (CHR6A). Bar is 10 uM. (C) The two chromosome 6s were extracted from panels A and B and aligned to show the BrdU incorporation and centromeric signals. (D) Pixel intensity profiles of BrdU incorporation and DAPI staining along the (6A) and (6B) chromosomes from panel C. The long (q) and short (p) arms of chromosome 6 are indicated. Bar is 2 uM. (E) BrdU quantification along 6A and 6B from panel D. Figure 5F shows the ratio of DNA synthesis into the two chromosome 6s in multiple cells by dividing the BrdU incorporation in 6B by the incorporation in 6A.

**Figure S7. Expression and asynchronous replication of *ASAR6*.** UCSC Genome Browser view of the *ASAR6* RNA-seq data, showing the reads from the plus and minus strands in separate tracks, from HTD114 nuclear ribo-minus RNA. We previously mapped the ~1.2 mb asynchronous replication domain associated with *ASAR6* as indicated {see (Donley et al. 2013)}. We note that the RNA-seq reads associated with *ASAR6* fulfill all of the criteria described for vlincRNAs {see (St Laurent et al. 2013; St Laurent et al. 2016)}. Two additional vlincRNAs (253 and 254) also map to the asynchronous replication domain. Also shown is the Repeat Masker Track.

**Table S1. Repetitive elements within vlinc273 (ASAR6-141).** The chromosome position, orientation, size and total bases occupied by LINE, Alu, and other repeats, from RepeatMasker are shown.

**Table S2.** **DNA oligonucleotides used for sgRNAs and PCR primers.** The DNA sequence of the oligonucleotides and the position on chromosome 6 of the sequences used for sgRNAs and PCR primers used to screen for deletions. Also shown are the heterozygous SNPs within the PCR products used to determine which allele was expressed and/or deleted following CRISPR/Cas9 expression.

**Table S3.** **Sequence junctions of the CRISPR/Cas9 deletion clones.** PCR products were generated using primers iii and viii on clones Δ6A(1-15-7), Δ6B(4-66-48), Δ6B(4-44-36), Δ6B(4-45-53) containing deletions of vlinc273, or i plus viii on clones Δ6A(R57-27), Δ6B(R57-37), Δ6A(R56-1) containing deletions of the entire vlinc cluster (vlinc271, vlinc1010, vlinc1011, vlinc1012, vlinc272, vlinc273), or i plus iv on clone Δ6B(L65-1) containing a deletion of vlinc271, vlinc1010, vlinc1011, vlinc1012, and vlinc272. PCR products were subjected to Sanger sequencing and the proximal and distal junctions were determined using BLAST. The deleted alleles were determined by loss of heterozygosity at 7 different SNPs (for examples see Figs. S1, S3 and S4).

**Table S4. Genomic location of vlincRNA genes.** The chromosome position, orientation and size of ASAR6 and the vlincRNA genes used in this study.
